# *In Situ* Polymerized Polyaniline in Redox-Active Metal–Organic Polyhedra for Supercapacitors

**DOI:** 10.1021/acsami.6c06826

**Published:** 2026-06-12

**Authors:** Yan-Ling Chang, Fuerkaiti Tayier, Cheng-Yan Hsieh, Shuhei Furukawa, Chung-Wei Kung

**Affiliations:** † Department of Chemical Engineering, 34912 National Cheng Kung University, Tainan City 70101, Taiwan; ‡ Institute for Integrated Cell-Material Sciences (WPI-iCeMS), 12918 Kyoto University, Yoshida, Sakyo-ku, Kyoto 606-8501, Japan; § Department of Synthetic Chemistry and Biological Chemistry, Graduate School of Engineering, Kyoto University, Katsura, Nishikyo-ku, Kyoto 615-8510, Japan; ∥ Program on Key Materials, Academy of Innovative Semiconductor and Sustainable Manufacturing, National Cheng Kung University, Tainan City 70101, Taiwan

**Keywords:** conducting polymer, energy storage, nanocomposite, pseudocapacitor, ruthenium-based MOP

## Abstract

The introduction of porosity into polyaniline (PANI) is key for enhancing its capacitive performance because both electrons and counterions need to migrate through the whole material during the charge–discharge process. Here, we demonstrated the incorporation of redox-active metal–organic polyhedra (MOPs) as intrinsic porous materials into PANI. Two redox-active and structurally stable ruthenium-based MOPs, **SO**_**3**_ **-RuMOP** and ***t*** **-Bu-RuMOP**, were employed as additives during the solution-phase oxidative polymerization of aniline. This approach produced two series of nanocomposites with various ratios between MOPs and PANI. Dynamic light scattering measurements were used to trace the hydrodynamic diameters of solid products formed during the polymerization process and to investigate the effect of different MOPs on the process. Electron microscopy and infrared spectroscopy were used to quantify the MOPs loading in each composite. Electrochemical measurements of pristine materials and all PANI@MOP composites were conducted in acidic aqueous electrolytes to study the electrochemistry of both MOPs and PANI in the composites. Composites with the optimal capacitive performance in both series, **PANI@SO**_**3**_ **-RuMOP** (1:0.035) and **PANI@** ***t*** **-Bu-RuMOP** (1:0.07), achieve specific capacitances of 416 ± 37 F/g and 396 ± 34 F/g at 0.5 mA/cm^2^, respectively. These capacitive performances significantly outperform the pristine PANI (347 ± 22 F/g). Both composites also exhibited better rate capability and higher long-term capacitance retention than pristine PANI. Findings here shed light on the use of redox-active MOPs as a minor additive during in situ polymerization to synthesize conducting polymers with enhanced capacitive performance for electrochemical energy storage devices.

## Graphical abstract

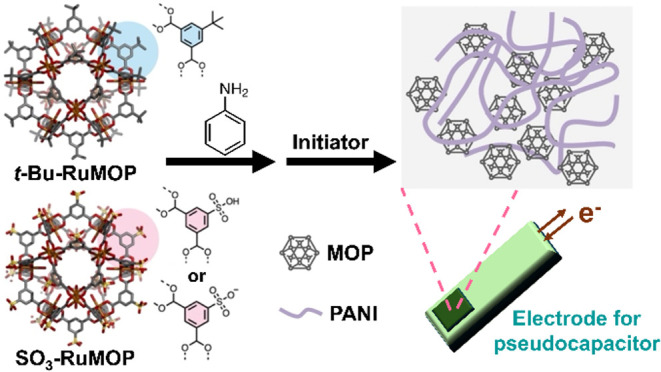

## Introduction

Future energy storage devices will require a higher power density and faster charging. Supercapacitors fulfill these requirements and provide power density beyond that of conventional batteries.^1^ Numerous materials, including metal oxides, conducting polymers, and carbon-based materials, have been investigated for supercapacitor applications.^2–5^ Among those, polyaniline (PANI) is especially attractive, owing to its high theoretical capacitance per unit mass, known as specific capacitance.^1,4,6,7^ The high specific capacitance of PANI relies on pseudocapacitance, which originates from its facile and reversible Faradaic processes.^8,9^ To achieve the highest specific capacitance of PANI as close as its theoretical value, redox-active units of PANI need to be more electrochemically addressable, *i.e*., accessible to both electrons and counterions during the charge–discharge process. Thus, extensive efforts have been made to synthesize PANI with a high external surface area or to prevent its aggregation.^10–12^

*In situ* polymerization of aniline in the presence of porous support with rigid and interconnected pores is an appealing strategy to obtain PANI with a large external surface area. Metal–organic frameworks (MOFs), crystalline solids with interconnected micropores,^13^ have shown a strong potential for the synthesis of PANI-based nanocomposites.^14–19^ The confinement of PANI inside the MOF pores suppresses the aggregation of PANI, and the residual pores of the rigid framework facilitate the mass transfer of counterions. Several recent studies reported the *in situ* polymerization of aniline within stable MOFs for supercapacitor applications.^16,20–22^ We recently demonstrated a water-stable two-dimensional MOF with sulfonate groups as a porous support during the polymerization of aniline.^22^ The negatively charged sulfonate groups attracted the positively charged aniline during the polymerization and promoted composite formation. PANI in this composite thus achieved improved specific capacitance compared to pristine PANI. However, ion transport inside MOF crystals limits the rate performance of confined PANI during charge–discharge processes. This concern becomes more significant when the MOF crystals have larger sizes. To further enhance the capacitive performance of PANI in these nanocomposites, it is necessary to introduce larger pores into the MOF,^23^ or reduce the crystal size of MOF.

Here, we focus on metal–organic polyhedra (MOPs), representing the smallest pore units of MOF crystals. MOPs are discrete cage-like porous molecules with an accessible intrinsic cavity.^24–27^ We hypothesized that *in situ* polymerization of aniline in the presence of fully dispersed MOPs would produce PANI with high spatial dispersion, which promotes faster ion transport during charge–discharge processes. Among various types of MOPs, ruthenium-based MOPs (RuMOPs) are especially attractive, since they are structurally stable in acidic aqueous solutions,^28,29^ required for aniline polymerization and for the electrochemical operation of PANI.^1,4,6,30,31^ In addition, the RuMOPs are redox-active,^29,32^ which should contribute to additional pseudocapacitance.

In this work, we synthesized two series of PANI@MOP nanocomposites with various PANI/MOP ratios. Two redox-active RuMOPs, [Ru_2_(SO_3_-bdc)­(HSO_3_-bdc)]_12_ (**SO**_**3**_ **-RuMOP**, SO_3_-bdc = 5-sulfonate-1,3-benzenedicarboxylate, HSO_3_-bdc = 5-sulfonic acid-1,3-benzenedicarboxylate) and [Ru_2_(*t*-Bu-bdc)_2_]_12_(BF_4_)_12_ (***t*** **-Bu-RuMOP**, *t*-Bu-bdc = 5-*tert-*butylbenzene-1,3-dicarboxylate), were introduced during the *in situ* polymerization of aniline (Figure 1). These RuMOPs possess the cuboctahedral geometry with the mixed-valence Ru_2_(II,III) paddlewheels as vertices and isophthalate derivatives as sides. **SO**_**3**_ **-RuMOP** features hydrophilic sulfonate moieties and disperses homogeneously in acidic aqueous solutions (Figure S1). Note that **SO**_**3**_ **-RuMOP** compensates for the positive charges of Ru_2_ paddlewheels by protonation of the sulfonate groups and does not require additional anions. On the other hand, ***t*** **-Bu-RuMOP** has hydrophobic *tert*-butyl groups and can aggregate in aqueous solution (Figure S2). Because of the presence of Ru_2_ paddlewheel units, ***t*** **-Bu-RuMOP** requires BF_4_^–^ anions to maintain charge neutrality. Both PANI and the MOPs show redox activity, so that PANI@MOP nanocomposites are expected to exhibit a higher capacitive performance than pristine PANI.

**Figure 1:**
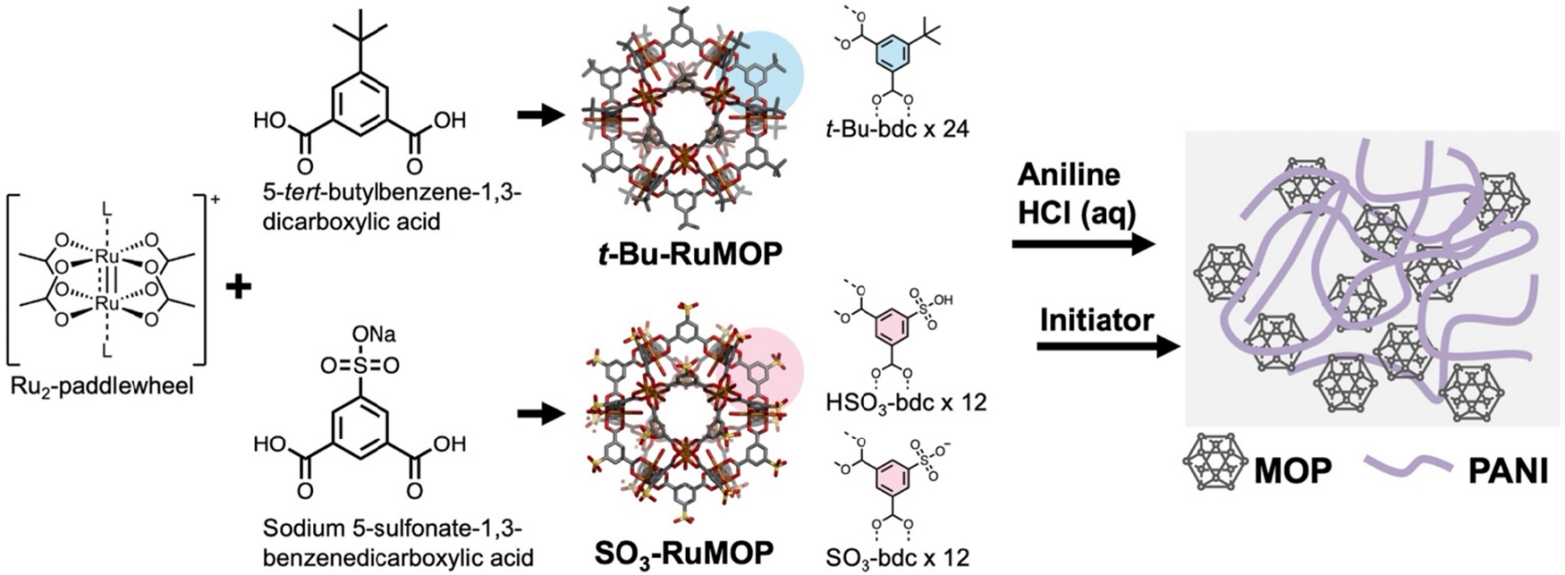
Synthesis of ruthenium-based MOPs (RuMOPs) and their use in the solution-phase *in situ* polymerization of aniline. The counteranion of Ru_2_-paddlewheel was omitted. The blue and pink organic ligands indicate the types and quantities of organic ligands in the corresponding MOPs. In the crystal structure of RuMOPs, C, O, Ru, and S are displayed in gray, red, brown, and yellow, respectively; hydrogen, solvent, and counteranions were omitted. *t*-Bu-bdc = 5-*tert*-butylbenzene-1,3-dicarboxylate; SO_3_-bdc = 5-sulfonate-1,3-benzenedicarboxylate; HSO_3_-bdc = 5-sulfonic acid-1,3-benzenedicarboxylate.

## Experimental Section

### Chemicals

Aniline (≥99.5%) and ammonium persulfate (APS, ≥98%) were purchased from Sigma-Aldrich. Hydrochloric acid (HCl, 36.5–38.0%) was supplied by J. T. Baker. Diethyl ether (≥99.8%) was purchased from Honeywell Fluka. Acetone (>99.0%) was purchased from UNI-ONWARD Corp., Ltd., Taiwan. *N*,*N*-Dimethylacetamide (DMA, ≥98.0%), *N*,*N*-dimethylformamide (DMF, ≥99.7%), fluoroboric acid 50 wt % solution in water (HBF_4_), tetrahydrofuran (THF, ≥98.0%), acetic acid (≥99.0%), and acetic anhydride (≥97.0%) were supplied by Nacalai Tesque, Inc. Lithium chloride (LiCl, ≥99.0%) was purchased from FUJIFILM Wako Chemicals. Ruthenium­(III) chloride hydrate (RuCl_3_·*n*H_2_O, 40 wt %) was purchased from TANAKA Precious Metals. Silver tetrafluoroborate (AgBF_4_, >98.0%) was purchased from Tokyo Chemical Industry Corp., Ltd., Japan. Information on the chemicals used for preparing inductively coupled plasma–optical emission spectrometry (ICP-OES) samples can be found in our recent work.^23^

### Synthesis of RuMOPs

***t*** **-Bu-RuMOP** and **SO**_**3**_ **-RuMOP** were synthesized using the reported methods with modifications.^29,33,34^ The starting cationic diruthenium complex, [Ru_2_(OAc)_4_(THF)_2_]­(BF_4_), was prepared by following a previously reported protocol.^35^ See the Supporting Information for detailed synthetic protocols.

The compound of ***t*** **-Bu-RuMOP** was synthesized by dissolving [Ru_2_(OAc)_4_(THF)_2_]­(BF_4_), *t*-Bu-H_2_bdc, and Na_2_CO_3_ in DMA, followed by heating at 120 °C to yield the corresponding single crystals of ***t*** **-Bu-RuMOP**. The crystals were suspended in methanol, and an aqueous solution of HBF_4_ was added. The precipitate obtained by centrifugation was dissolved in DMF and poured into diethyl ether to give yellowish-brown amorphous solids. The obtained solids were dried under ambient conditions to yield the final MOP powders. This powder dissolves in various organic solvents and was used for all experiments.

The compound of **SO**_**3**_ **-RuMOP** was synthesized by dissolving [Ru_2_(OAc)_4_(THF)_2_]­(BF_4_) and sodium 5-sulfonate-1,3-benzenedicarboxylic acid (NaSO_3_–H_2_bdc) in a DMA:H_2_O (9:1) mixture. The mixture was heated at 120 °C for 16 h to yield red-brown crystals of **SO**_**3**_ **-RuMOP**. The crystals were suspended in methanol, and triflic acid (HOTf) was added to obtain a red-brown solution. **SO**_**3**_ **-RuMOP** was precipitated with diethyl ether to give a brown powder. The powder was dried under ambient conditions to yield the final MOP powder. This powder was used for all experiments.

### Polymerization of Aniline

The aniline was polymerized in the presence of RuMOP using a procedure similar to our previous work.^22,23^ Detailed procedures can be found in Supporting Information. First, **SO**_**3**_ **-RuMOP** solution or ***t*** **-Bu-RuMOP** suspension was prepared with various solid concentrations. An aqueous HCl solution containing aniline was added to the prepared **SO**_**3**_ **-RuMOP** solution or ***t*** **-Bu-RuMOP** suspension. An aqueous solution of APS was then added under continuous stirring. The reaction mixture was stirred overnight to complete polymerization. The obtained solid was washed with aqueous HCl solution, acetone, and diethyl ether. After removal of the final supernatant, the solid was dried under vacuum at room temperature overnight to obtain the final nanocomposite. The obtained nanocomposites with **SO**_**3**_ **-RuMOP** and ***t*** **-Bu-RuMOP** were named as “**PANI@SO**_**3**_ **-RuMOP** (1:*X*)” and “**PANI@** ***t*** **-Bu-RuMOP** (1:*X*),” respectively, where *X* corresponds to the mass ratio between the MOP and aniline added during the synthesis (*X* = 1.5, 0.25, 0.07, 0.035, and 0.018). As a comparison, the pristine PANI was synthesized with the same procedure except that the initial RuMOP solution or suspension was replaced by an equal volume of H_2_O.

### Preparation of Modified Electrodes

The drop-casting process with the use of fluorine-doped tin oxide (FTO) glass substrates (exposed area = 0.25 cm^2^) was employed to fabricate modified electrodes of all materials. Detailed protocols regarding the preparation of FTO substrates can be found in our previous studies.^23,36^ Herein, 3.0 mg of the pristine PANI, nanocomposite, or pristine ***t*** **-Bu-RuMOP** was dispersed in 1.25 mL of acetone by ultrasonication. Thereafter, 6 μL of the suspension was drop-cast onto the FTO substrate, and the casting process was repeated three times in order to achieve full coverage. The same casting procedure for PANI has been employed in our previous work.^23^

### Instrumentation

Scanning electron microscopic (SEM) images were collected by SU-8230 or SU5000 (Hitachi). Transmission electron microscopic (TEM) images and the corresponding energy-dispersive X-ray spectroscopic (EDS) data were collected by JEM-2200FS (JEOL). Fourier transform infrared (FTIR) spectra were collected by a Nicolet 6700 (Thermo Scientific), under the transmission sampling mode at a resolution of 4 cm^–1^. The sample was ground with KBr, followed by pelletization before FTIR measurements. X-ray photoelectron spectroscopy (XPS) was measured by a Theta Probe (Thermo Scientific). Correction was performed on all XPS data by referencing the C 1s peak to 284.7 eV. N_2_ sorption isotherm was measured by using BELSORP-mini X (MicrotracBel). The samples were activated at 120 °C for 16 h under vacuum before measurement. Dynamic light scattering (DLS) and ζ-potential measurements were performed on a Zetasizer Nano ZS (Malvern Instruments). For ICP-OES quantification, 3.0 mg of MOP or MOP-PANI nanocomposite was accurately weighed in a microwave vial, and the microwave digestion was performed to prepare 40 mL of the sample solution; see the detailed protocol reported in our previous study.^23^ A JY 2000-2 (Horiba Scientific) was used for all ICP-OES measurements in order to quantify ruthenium in each sample solution.

DLS measurements were performed to trace the polymerization processes. A 1 mL suspension was collected from the reaction mixture under stirring at 1, 3, 6, and 24 h. The hydrodynamic diameters of PANI and the nanocomposites were measured at each time point. The mean and standard deviation of the hydrodynamic diameters were based on three measurements obtained from separate polymerization batches. ζ-potential measurements were conducted in H_2_O and diluted HCl (2 mM) to maintain a low ionic strength suitable for electrophoretic analysis. The reported ζ-potential was calculated by the Zetasizer from the measured electrophoretic mobility. This value corresponds to the electrostatic potential at the shear plane located between the diffuse layer and the bulk solution.

Cyclic voltammetry (CV) and galvanostatic charge–discharge (GCD) data were collected by a CHI6273E (CH Instruments). Aqueous electrolytes containing 0.2 M HCl, which is the same as the solution used for washing after the synthesis, were used for all electrochemical measurements. A three-electrode setup was used for all electrochemical tests, with the FTO-based electrode, a platinum foil, and Ag/AgCl/NaCl (3 M) as the working electrode, counter electrode, and reference electrode, respectively.

For characterizing the material after the electrochemical measurement, a modified electrode with **PANI@SO**_**3**_ **-RuMOP** (1:1.5) after 20 cycles of CV scans between −0.2 V and +0.8 V at 25 mV/s was rinsed with water three times. After further rinsing with acetone three times, the composite thin film was dried at room temperature before further characterization.

## Results and Discussion

### Effect of MOPs on the Polymerization Process

ζ-Potential was used to study the interactions between RuMOPs and aniline monomers during polymerization. The ζ-potential of aniline in aqueous HCl solution was measured as +4.25 ± 0.39 mV. This positive value indicates that the aniline monomers remain positively charged under the acidic polymerization conditions. In H_2_O, the ζ-potentials of **SO**_**3**_ **-RuMOP** and ***t*** **-Bu-RuMOP** were measured as −28.5 ± 1.2 mV and +41.2 ± 5.4 mV, respectively (Figure S3a,d). The positive ζ-potential of ***t*** **-Bu-RuMOP** results from the positively charged Ru_2_ paddlewheel moieties in its structure. In **SO**_**3**_ **-RuMOP**, the surface charge is dominated by 24 sulfonate groups and results in an overall negative ζ-potential. Under the aqueous HCl conditions used for polymerization, the ζ-potentials of **SO**_**3**_ **-RuMOP** and ***t*** **-Bu-RuMOP** shift to −16.8 ± 1.6 mV and +15.1 ± 0.7 mV, respectively (Figure S3b,e). After 6 h, the ζ-potentials of **SO**_**3**_ **-RuMOP** and ***t*** **-Bu-RuMOP** reached −11.2 ± 0.8 mV and −12.9 ± 0.8 mV, respectively (Figure S3c,f). This progressive negative shift of ζ-potentials for ***t*** **-Bu-RuMOP** is attributed to chloride-rich acidic conditions. The first chloride ion forms an ion pair with the Ru paddlewheel and neutralizes its positive charge. This process replaces the BF_4_^–^ counterion in ***t*** **-Bu-RuMOP**. Over time, a second chloride ion forms an ion pair at the axial sites of the Ru_2_ paddlewheel. Each Ru_2_ paddlewheel then associates with two chloride anions and carries a net negative charge.^37^ Both RuMOPs therefore exhibit negative ζ-potentials under long-term exposure to aqueous HCl solutions. The electrostatic interaction between negatively charged MOPs and positively charged aniline monomers facilitates the polymerization of aniline near the MOP surface. It should be noticed that the ***t*** **-Bu-RuMOP** can only generate a suspension in the aqueous HCl (Figure S2), while the **SO**_**3**_ **-RuMOP** was fully dispersed into a homogeneous solution with a hydrodynamic diameter less than 3 nm (Figure S1). To probe the effect of MOPs on the polymerization process, reaction mixtures collected at different polymerization times were analyzed by DLS.

DLS measurements were conducted for PANI, **PANI@SO**_**3**_ **-RuMOP** (1:*X*), and **PANI@** ***t*** **-Bu-RuMOP** (1:*X*, *X* = 1.5, 0.25, 0.07, and 0.035). The hydrodynamic diameters of the PANI and the nanocomposites were measured at 1, 3, 6, and 24 h. At each time point, three replicate synthetic batches were prepared for measurement to obtain the mean and standard deviation (see Figures 2, S4, and S5).

**Figure 2:**
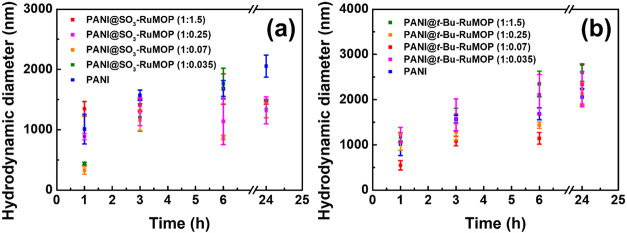
DLS results of suspensions collected during the synthesis of PANI and various nanocomposites with (a) **SO**_**3**_ **-RuMOP** and (b) ***t*** **-Bu-RuMOP**.

The hydrodynamic diameters of PANI were 1010 ± 246 nm at 1 h and increased to 2054 ± 184 nm at 24 h (Figure 2). **PANI@SO**_**3**_ **-RuMOP** (1:*X*) showed a similar increasing trend. The minimum hydrodynamic diameters of **PANI@SO**_**3**_ **-RuMOP** (1:*X*) were 326 ± 63 nm at 1 h and 1324 ± 121 nm at 24 h (Figures 2a and S4i–l). Compared to PANI, the hydrodynamic diameter of **PANI@SO**_**3**_ **-RuMOP** (1:*X*) remains smaller at most time points. **PANI@** ***t*** **-Bu-RuMOP** (1:*X*) with minimum sizes showed hydrodynamic diameters of 547 ± 102 nm at 1 h and 2333 ± 433 nm at 24 h (Figures 2b and S5i–l). These hydrodynamic diameter values of **PANI@** ***t*** **-Bu-RuMOP** are comparable to the values of PANI (Figure S5).

The effect of the mass ratio between MOP and aniline on the hydrodynamic diameter was also investigated. DLS measurements covered mass ratios from *X* = 0.035 to *X* = 1.5. An increase in *X* indicated that more RuMOP was added to the polymerization reaction. At 24 h, the hydrodynamic diameters of **PANI@SO**_**3**_ **-RuMOP** (1:*X*) at *X* = 0.035, *X* = 0.07, *X* = 0.25, and *X* = 1.5 were 1337 ± 140, 1324 ± 121, 1321 ± 224, and 1466 ± 41 nm, respectively. For **PANI@** ***t*** **-Bu-RuMOP** (1:*X*) at *X* = 0.035, *X* = 0.07, *X* = 0.25, and *X* = 1.5, the hydrodynamic diameters were 2230 ± 386, 2333 ± 433, 2130 ± 254, and 2598 ± 196 nm, respectively. At all mass ratios, **PANI@SO**_**3**_ **RuMOP** (1:*X*) showed a smaller hydrodynamic diameter than PANI at the same reaction time (Figure 2a). For **PANI@** ***t*** **-Bu-RuMOP** (1:*X*), the distribution range of the hydrodynamic diameter remained comparable to that of PANI (Figure 2b).

The above-mentioned results indicate that the hydrodynamic diameter of the nanocomposite is controlled by the type of RuMOP, with the solubility of the RuMOP playing a dominant role. **SO**_**3**_ **-RuMOP** dissolves fully and exists in aqueous HCl as discrete molecular cages. In contrast, ***t*** **-Bu-RuMOP** forms a suspension with aggregated MOPs. As a result, **PANI@SO**_**3**_ **-RuMOP** shows a smaller particle size at 24 h (Figure S6). This effect is most likely due to suppressed PANI aggregation, as observed before in MOF-based systems.^20–22^ The mass ratio has no significant effect on the hydrodynamic diameter of the final nanocomposite at 24 h. The only exception was **PANI@** ***t*** **-Bu-RuMOP** (1:1.5), where an increase in the RuMOP content led to a larger hydrodynamic diameter, likely due to the combined effect of low solubility and high RuMOP content.

### Characterizations of Materials

SEM images were first collected to examine the surface morphologies of each material. As shown in low-magnification images in Figure S7, **PANI@SO**_**3**_ **-RuMOP** (1:*X*) contains composite particles and the pristine PANI polymeric substrate. The pristine PANI is particularly presented in composites with low **SO**_**3**_ **-RuMOP** loading, including *X* = 0.018, 1:0.035, and 1:0.07 (Figure S7c–e). High-magnification SEM images were collected for **SO**_**3**_ **-RuMOP**, four **PANI@SO**_**3**_ **-RuMOP** (1:*X*) composites, and pristine PANI, with a particular focus on those composite particles. As revealed in Figure 3a,f, respectively, the pristine **SO**_**3**_ **-RuMOP** is composed of nanoparticles with sizes of around 50–70 nm, and the pristine PANI consists of one-dimensional nanofibers. For the four composites, large particles possessing sizes ranging from 100 to 200 nm with polymeric rough surfaces can be found in their SEM images (Figure 3b–e). These rough particles should be composed of aggregates of **SO**_**3**_ **-RuMOP** interconnected by the PANI solid, generated during the *in situ* polymerization of aniline in the presence of fully dispersed MOPs. On the other hand, ***t*** **-Bu-RuMOP** is composed of large octahedral crystals with a size of around 500 nm (Figures S8a and S9a). With the use of ***t*** **-Bu-RuMOP** as the additive during the polymerization, as shown in Figure S8, both PANI nanofibers and large octahedral crystals of MOPs can be found in the SEM images of the resulting composites. The corresponding high-magnification SEM images in Figure S9 show that the octahedral crystals found in all ***t*** **-Bu-RuMOP**-based composites possess similar polymeric rough surfaces, clearly indicating that the polymerization of aniline occurred on the surface of MOP particles during the synthesis.

**Figure 3:**
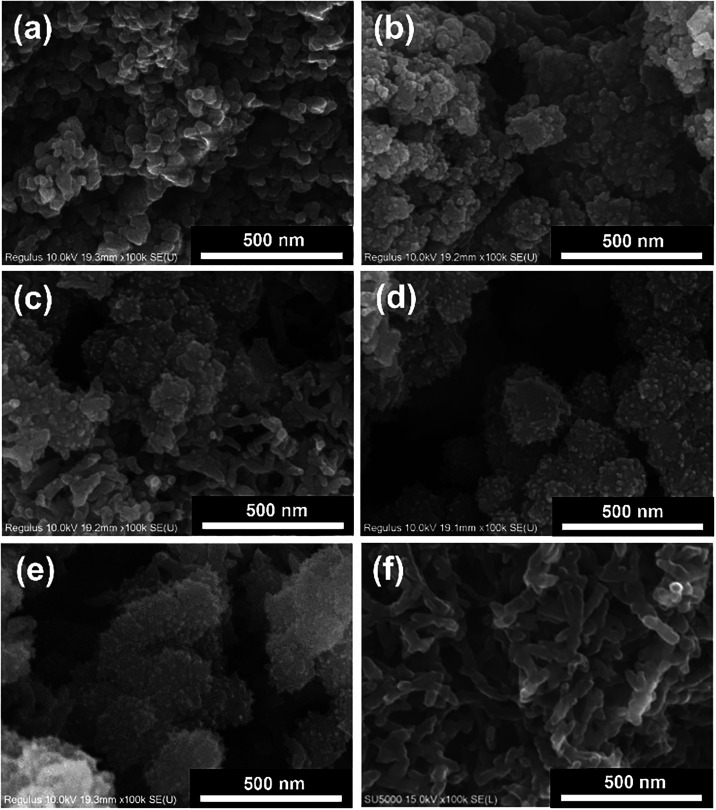
SEM images of (a) **SO**_**3**_ **-RuMOP**, (b) **PANI@SO**_**3**_ **-RuMOP** (1:1.5), (c) **PANI@SO**_**3**_ **-RuMOP** (1:0.07), (d) **PANI@SO**_**3**_ **-RuMOP** (1:0.035), (e) **PANI@SO**_**3**_ **-RuMOP** (1:0.018), and (f) PANI, collected at a high magnification.

TEM measurements coupled with energy-dispersive X-ray spectroscopy (EDS) elemental mapping were then performed to probe the spatial distributions of elements in representative materials. From Figure S10, it can be seen that **SO**_**3**_ **-RuMOP** is composed of nanoparticles, with uniform distributions of both sulfur and ruthenium in them. On the other hand, PANI consists of nanofibers with uniform distributions of both nitrogen and chlorine; the chlorine should originate from chloride ions, which serve as the dopant of PANI synthesized from HCl. We then collected TEM and EDS data for two representative MOP-PANI nanocomposites with high MOP loadings, **PANI@SO**_**3**_ **-RuMOP** (1:1.5) and **PANI@** ***t*** **-Bu-RuMOP** (1:1.5), and the results are shown in Figure 4. Consistent with its SEM image, **PANI@SO**_**3**_ **-RuMOP** (1:1.5) is composed of small particles, and uniform distributions of both ruthenium and nitrogen can be found in these particles (Figure 4a–c). For **PANI@** ***t*** **-Bu-RuMOP** (1:1.5), large octahedral crystals can be found in its TEM image (Figure 4d), and EDS mapping data suggest that both ruthenium and nitrogen are uniformly present within such large crystals (Figure 4e,f). Similar uniform distributions of elements can also be found in the nanocomposite with a low loading of MOPs (Figure S11). Findings here indicate that, during the *in situ* polymerization in the presence of MOPs, owing to the difference in ζ-potentials of aniline and both MOPs, PANI could be generated either on the surface of dispersed MOPs or inside the MOPs to form the composite particles with a heterostructural interface. Even though ***t*** **-Bu-RuMOP** possesses much larger particle sizes, the polymerization of aniline could still occur uniformly to cover such large MOP particles.

**Figure 4:**
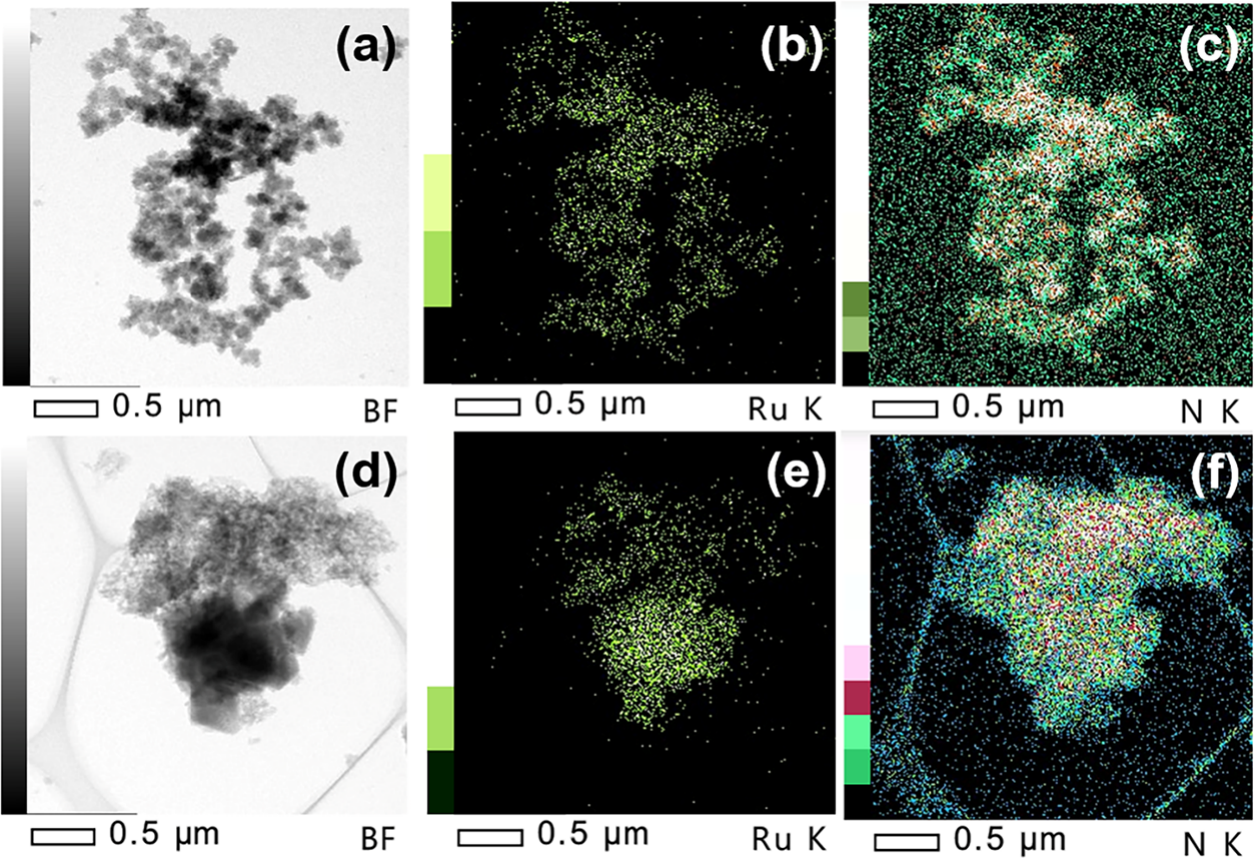
TEM images of (a) **PANI@SO**_**3**_ **-RuMOP** (1:1.5) and (d) **PANI@** ***t*** **-Bu-RuMOP** (1:1.5). EDS mapping signals of (b) ruthenium and (c) nitrogen, collected from the region shown in (a). EDS mapping signals of (e) ruthenium and (f) nitrogen, collected from the region shown in (d).

FTIR measurements were performed to characterize all materials (Figure 5). For PANI, characteristic peaks for the C=C stretching of quinoid at 1560 cm^–1^, C=C stretching of benzene at 1490 cm^–1^, C–N and C–N^+^ stretching at 1303 cm^–1^, C–H bending of quinoid at 1146 cm^–1^, and C–H out-of-plane bending on the aromatic ring of PANI at around 805 cm^–1^ can be observed in its spectrum.^16,22^ As shown in both Figure 5a,b, these peaks can be clearly found in spectra of all the eight nanocomposites with **SO**_**3**_ **-RuMOP** or ***t*** **-Bu-RuMOP**, which confirms the presence of PANI in these composite materials. On the other hand, the FTIR spectrum of **SO**_**3**_ **-RuMOP** in Figure 5a shows three characteristic peaks at 1437, 1378, and 1040 cm^–1^. The peaks at 1437 and 1378 cm^–1^ correspond to the asymmetric stretching *v*_asym_(O–C–O) and symmetric stretching *v*_sym_(O–C–O), respectively, of bridging carboxylates in RuMOPs.^29^ The peak at 1040 cm^–1^ corresponds to the symmetric stretching of sulfonate groups.^38^ The FTIR spectrum of ***t*** **-Bu-RuMOP** in Figure 5b shows characteristic peaks at 1437 and 1378 cm^–1^ from *v*_asym_(O–C–O) and *v*_sym_(O–C–O), respectively.^29^ A broad absorption band appearing around 1050 cm^–1^ corresponds to the stretching vibration of BF_4_^–^ counteranions. Both **SO**_**3**_ **-RuMOP** and ***t*** **-Bu-RuMOP** exhibit mixed-valence Ru_2_(II,III) paddlewheel moieties. This mixed-valence state is indicated by the separation (Δ*ν*) between *ν*_sym_(O–C–O) and *ν*_asym_(O–C–O), Δ*ν* = *ν*_asym_(O–C–O) – *ν*_sym_(O–C–O). The Δν values are 59 cm^–1^ for **SO**_**3**_ **-RuMOP** and 57 cm^–1^ for ***t*** **-Bu-RuMOP**. These values fall within the reported range for Ru_2_(II,III) paddlewheels of 35–144 cm^–1^ and agree with previous studies.^28,29^

**Figure 5:**
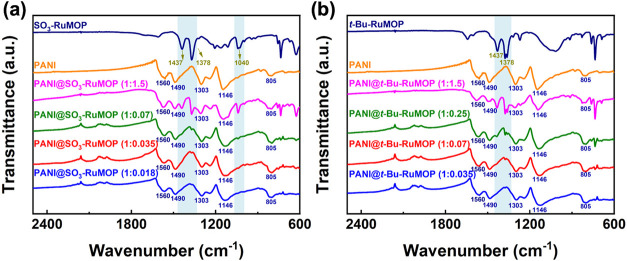
FTIR spectra of MOPs and nanocomposites with (a) **SO**_**3**_ **-RuMOP** and (b) ***t*** **-Bu-RuMOP** with different PANI-to-MOP ratios. The spectrum of pristine PANI is also shown. Blue lines indicate the peaks associated with MOPs.

For **PANI@SO**_**3**_ **-RuMOP** (1:*X*), the three characteristic peaks of **SO**_**3**_ **-RuMOP** appear in the spectrum of **PANI@SO**_**3**_ **-RuMOP** (1:1.5). This observation confirms the presence of **SO**_**3**_ **-RuMOP** in the nanocomposite. The spectra of the other three **PANI@SO**_**3**_ **-RuMOP** (1:*X*) samples show weak MOP signals due to their low **SO**_**3**_ **-RuMOP** loadings. The ICP-OES results discussed later support these spectral results. For **PANI@** ***t*** **-Bu-RuMOP** (1:*X*), the characteristic *v*_asym_(O–C–O) and *v*_sym_(O–C–O) peaks of ***t*** **-Bu-RuMOP** appear in the spectra of **PANI@** ***t*** **-Bu-RuMOP** (1:1.5) and **PANI@** ***t*** **-Bu-RuMOP** (1:0.25). The absence of the BF_4_^–^ band near 1050 cm^–1^ indicates the replacement of BF_4_^–^ counteranions by chloride anions in aqueous HCl. As a result, the FTIR spectra demonstrate that a series of **PANI@RuMOP** nanocomposites were obtained without significant changes to the organic structure of PANI or the metal valence state of the RuMOPs.

To quantify the loading of MOPs in each nanocomposite, 3.0 mg of each material was accurately weighed, followed by digestion to prepare the sample for ICP-OES measurements. From the ruthenium concentration in each sample, the mass loading of MOPs in the nanocomposite was estimated; a similar method was used for MOF-PANI nanocomposites in our previous studies.^22,23^ ICP-OES results for **SO**_**3**_ **-RuMOP**-based and ***t*** **-Bu-RuMOP**-based materials are summarized in Tables S1 and S2, respectively. Mass loadings of MOPs in **PANI@SO**_**3**_ **-RuMOP** (1:1.5), **PANI@SO**_**3**_ **-RuMOP** (1:0.07), **PANI@SO**_**3**_ **-RuMOP** (1:0.035), and **PANI@SO**_**3**_ **-RuMOP** (1:0.018) were found as 66.9, 10.8, 5.6, and 2.7 wt %, respectively. For **PANI@** ***t*** **-Bu-RuMOP** (1:1.5), **PANI@** ***t*** **-Bu-RuMOP** (1:0.25), **PANI@** ***t*** **-Bu-RuMOP** (1:0.07), and **PANI@** ***t*** **-Bu-RuMOP** (1:0.035), their mass loadings of MOPs are 71.2, 23.0, 9.2, and 3.4 wt %, respectively. In general, mass loadings of MOPs in all nanocomposites follow the trend in the amounts of MOPs added during *in situ* polymerization.

XPS spectra in the region of Ru 3p were collected to probe the valence states of ruthenium in the pristine **SO**_**3**_ **-RuMOP** and the representative composite, **PANI@SO**_**3**_ **-RuMOP** (1:1.5). As shown in Figure S12a, the **SO**_**3**_ **-RuMOP** shows a single peak centered at 464.0 eV in its XPS spectrum, which is consistent with the characteristics of Ru­(III)-based compounds reported in the literature.^39,40^ For the **PANI@SO**_**3**_ **-RuMOP** (1:1.5), the same peak centered at 464.0 eV can also be observed in its XPS spectrum (Figure S12b). Findings here suggest that in both the pristine **SO**_**3**_ **-RuMOP** and the nanocomposite, their ruthenium sites are mainly Ru­(III). A thin film of **PANI@SO**_**3**_ **-RuMOP** (1:1.5) was then deposited on an FTO-based electrode for performing 20 cycles of CV measurement, in an aqueous electrolyte containing 0.2 M of HCl. The thin film after the electrochemical measurement was collected and subjected to XPS measurements. As revealed in Figure S12c, a single characteristic peak can still be observed in the obtained XPS spectrum in the region of Ru 3p, with its center shifting to 463.6 eV; this peak location should still be associated with Ru­(III).^39^ But it should be noticed that the valence state of Ru­(III)/Ru­(II) is difficult to distinguishable solely according to Ru 3p spectra.^41^ Thus, XPS spectra of both **PANI@SO**_**3**_ **-RuMOP** (1:1.5) samples before and after the CV measurement were collected in regions of Ru 3d and N 1s. Since regions of Ru 3d and C 1s are partially overlapping, peaks from both regions were deconvoluted. As shown in Figure 6a,b, C 1s peaks from both the organic moieties of RuMOP and PANI can be identified in both samples.^42^ Before the electrochemical measurement, only one single peak in the region of Ru 3d centered at 282.4 eV can be observed, corresponding to Ru­(III).^39,43–45^ After the CV measurement, as revealed in Figure 6b, two Ru 3d peaks at 282.6 and 281.4 eV, associated with Ru­(III) and Ru­(II), respectively,^39,43–45^ can both be deconvoluted. Results here clearly indicate that a quite significant proportion of ruthenium sites in **PANI@SO**_**3**_ **-RuMOP** (1:1.5) are electrochemically active. In addition, peaks of C=N–C, N–H, and −N^+^– from PANI can be deconvoluted in both N 1s spectra shown in Figure 6c,d.^46,47^ A significant decrease in the intensity of both peaks for C=N–C and −N^+^– and increase in the peak intensity for N–H can be observed after the electrochemical measurement, which also indicates that PANI in this composite is redox-active during the electrochemical operation; also see discussion on electrochemical results in the later section.

**Figure 6:**
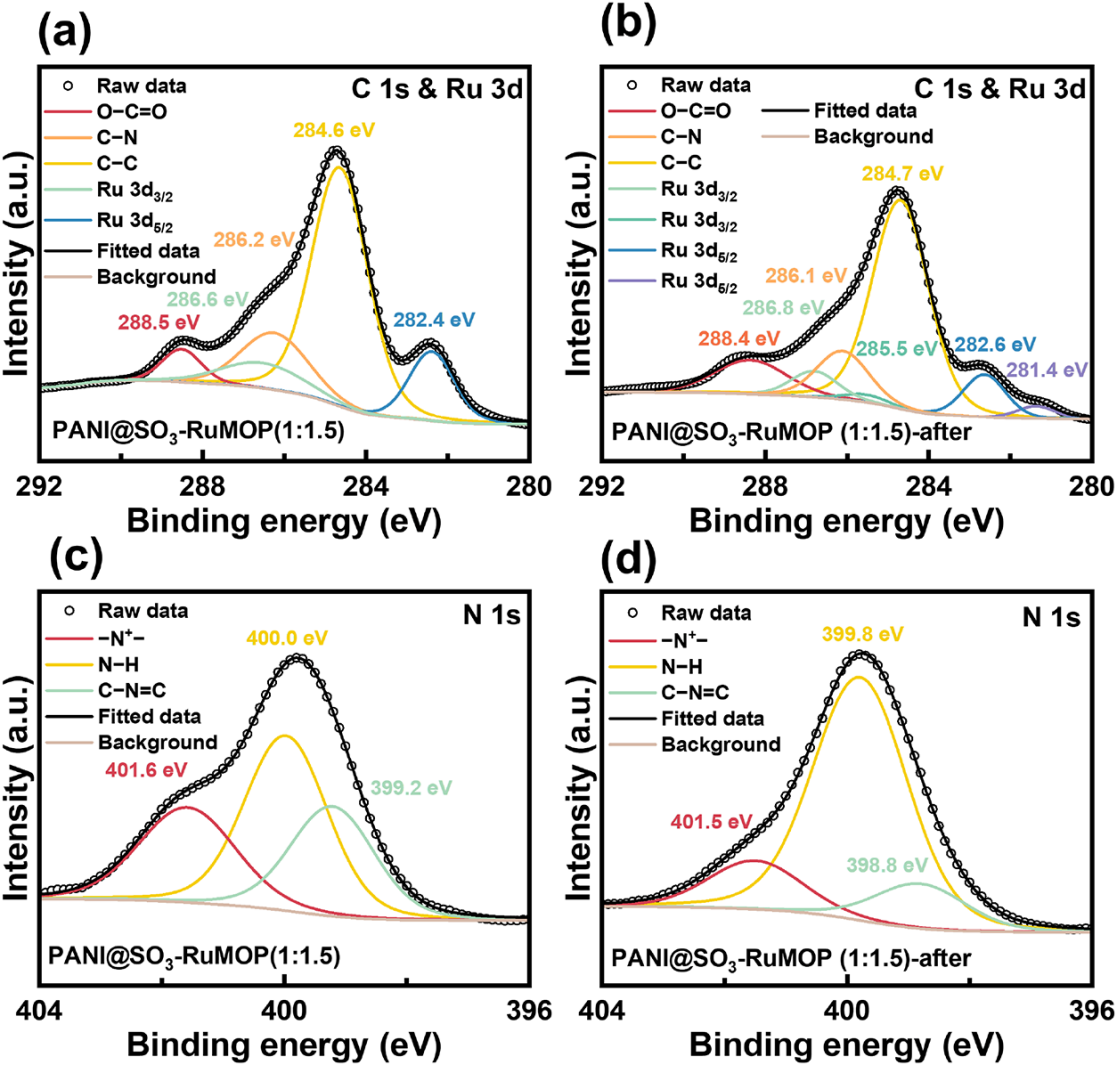
XPS spectra of (a, c) **PANI@SO**_**3**_ **-RuMOP** (1:1.5), and (b, d) thin film of **PANI@SO**_**3**_ **-RuMOP** (1:1.5) after 20 cycles of CV scans, collected in the regions of Ru 3d, C 1s, and N 1s.

### Porous properties

The N_2_ sorption isotherms for **SO**_**3**_ **-RuMOP**, ***t*** **-Bu-RuMOP**, **PANI@SO**_**3**_ **-RuMOP** (1:1.5), and **PANI@** ***t*** **-Bu-RuMOP** (1:1.5) were measured at 77 K to evaluate the porosities of composites (Figure 7). Compared with the best-performing composites, the higher MOP loading in PANI@MOP (1:1.5) makes it a suitable sample for investigating changes in N_2_ uptake. Both the pristine MOP and the nanocomposites exhibit a sharp uptake of N_2_ in the lower relative pressure region, indicating the presence of microporosity. At *P*/*P*_0_ = 0.1, the N_2_ sorption of **SO**_**3**_ **-RuMOP**, ***t*** **-Bu-RuMOP**, **PANI@SO**_**3**_ **-RuMOP** (1:1.5), and **PANI@** ***t*** **-Bu-RuMOP** (1:1.5) were 27, 126, 27, and 32 cm^3^/g, respectively. The N_2_ sorption of the ***t*** **-Bu-RuMOP** is consistent with previous reports, and the **SO**_**3**_ **-RuMOP** is in agreement with previously reported Rh_2_ paddlewheel-based analogs.^29,33^ Compared to other samples, ***t*** **-Bu-RuMOP** exhibits a high N_2_ sorption value of 126 cm^3^/g. This high N_2_ sorption is because of the interaction between the *t*-Bu groups, which generate extrinsic porosity that allows nitrogen to diffuse through and access all the intrinsic cavities of MOPs. On the other hand, during sorption measurement, **SO**_**3**_ **-RuMOP** was closely packed into amorphous aggregates and exhibited low nitrogen adsorption at 77 K.^48^ The introduction of PANI into the MOP significantly decreased the N_2_ sorption of **PANI@** ***t*** **-Bu-RuMOP** (1:1.5). This significant decrease is attributed to PANI blocking the extrinsic pore of ***t*** **-Bu-RuMOP**, thus limiting the accessibility of nitrogen into the intrinsic cavities of ***t*** **-Bu-RuMOP**. Previous studies on MOF-based PANI composites have also reported a similar phenomenon, with the MOF-based PANI composites exhibiting lower sorption compared to pristine MOF.^20^ In contrast, the N_2_ sorption of both **SO**_**3**_ **-RuMOP** and **PANI@SO**_**3**_ **-RuMOP** is low, as nitrogen diffusion is limited in both **SO**_**3**_ **-RuMOP** and **PANI@SO**_**3**_ **-RuMOP**. The Brunauer–Emmett–Teller (BET) specific surface area was calculated by the BETSI program to be 110, 508, 108, and 128 m^2^/g (Figures S19–S22) for **SO**_**3**_ **-RuMOP**, ***t*** **-Bu-RuMOP**, **PANI@SO**_**3**_ **-RuMOP** (1:1.5), and **PANI@** ***t*** **-Bu-RuMOP** (1:1.5), respectively.^49^ The finding here suggests that in the PANI@MOP composites, the presence of PANI suppresses the accessibility of N_2_ to the intrinsic cavity of MOP.

**Figure 7:**
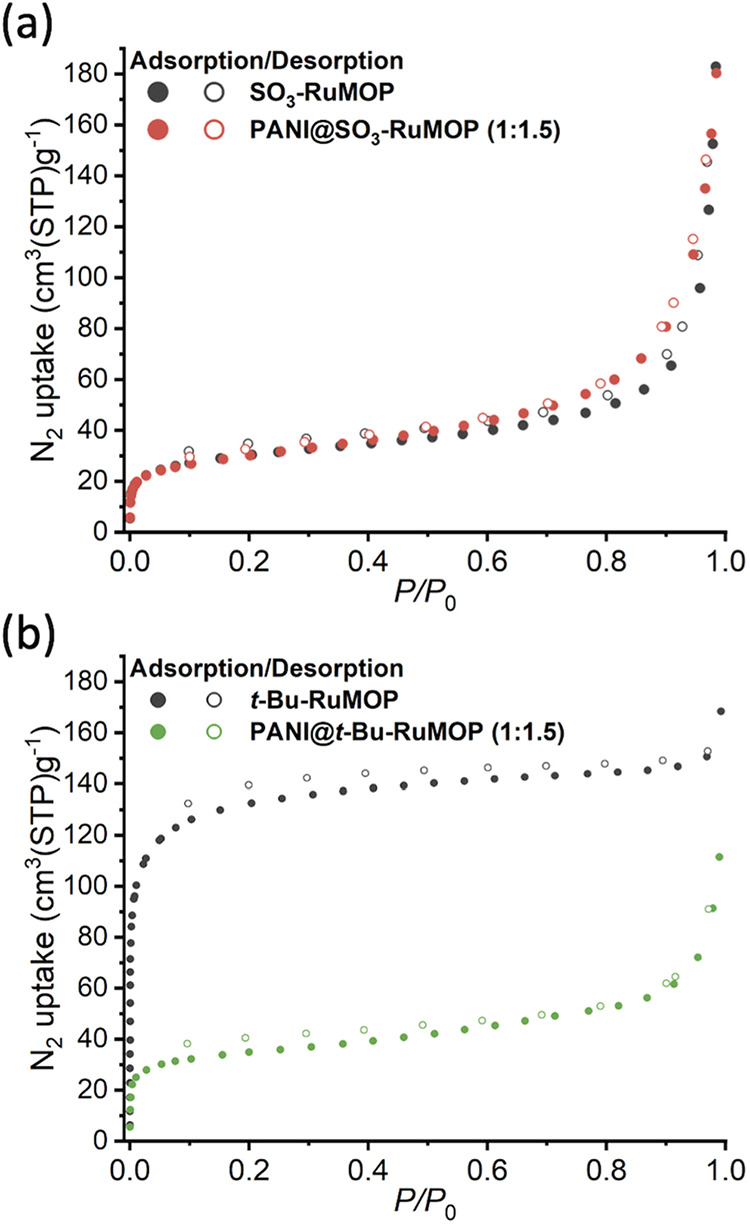
N_2_ sorption isotherm measured at 77 K. (a) **SO**_**3**_ **-RuMOP** (gray) and **PANI@SO**_**3**_ **-RuMOP** (1:1.5) (red); (b) ***t*** **-Bu-RuMOP** (gray) and **PANI@** ***t*** **-Bu-RuMOP** (1:1.5) (green). Samples were activated at 120 °C for 16 h before measurements.

### Electrochemical performance

Thin films of the pristine MOPs, pristine PANI, and all nanocomposites were then fabricated on FTO-based conducting electrodes to investigate their electrochemical behavior and capacitive performance. Aqueous solutions containing 0.2 M HCl, the same environment used during the *in situ* polymerization for synthesizing all nanocomposites and PANI, were employed as electrolytes for all electrochemical tests.^22^ It should be noted that since the **SO**_**3**_ **-RuMOP** is fully dispersible in aqueous solutions, immersing the pristine **SO**_**3**_ **-RuMOP** thin film into such an aqueous electrolyte results in the dissolution of the thin film. Therefore, for the electrochemical characterization of pristine MOPs, we only tested the thin film of ***t*** **-Bu-RuMOP**. As shown in Figure 8a, one set of reversible redox peaks is centered at around +0.08 V vs Ag/AgCl/NaCl (3 M) can be observed in the CV curve of the ***t*** **-Bu-RuMOP** thin film, and such Faradaic responses should be attributed to the reversible redox reaction of the ***t*** **-Bu-RuMOP** between Ru­(III) and Ru­(II).^29^ Although the redox peaks of MOPs are clearly observed, their current responses become negligible when compared with those of the pristine PANI and PANI-based nanocomposites (Figure 8b). The pristine PANI shows two sets of broad and reversible redox peaks in its CV curve, centered at around +0.21 V and +0.48 V, respectively. These peaks should originate from the reversible reactions between leucoemeraldine, emeraldine, and pernigraniline states of PANI, consistent with the previously reported electrochemical characteristics of PANI in acidic electrolytes.^6,22,23,50^ Broad peaks in the CV curve with a large enclosed area also imply the pseudocapacitive characteristic of PANI.^8,51^ For **PANI@** ***t*** **-Bu-RuMOP** (1:1.5), which possesses around 70 wt % of MOPs and 30 wt % of PANI, its CV curve shows much smaller redox peaks from PANI. It should be noted that in the CV curve of the **PANI@** ***t*** **-Bu-RuMOP** (1:1.5) thin film, redox peaks centered at +0.21 V exhibit much larger responses compared to those centered at +0.48 V. As revealed in Figure 8b, such a difference in the peak ratio becomes less significant with decreasing mass loading of MOPs in the nanocomposite. Since the ***t*** **-Bu-RuMOP** has redox peaks located at similar potentials to the first set of redox peaks of PANI, observations here imply that the Faradaic response of the ***t*** **-Bu-RuMOP** in the nanocomposite may overlap with that of PANI and thus amplify the response of the redox peaks centered at around +0.21 V. Moreover, the enclosed area of redox peaks centered at around +0.21 V in the CV curve of **PANI@** ***t*** **-Bu-RuMOP** (1:1.5) is more than 1 order of magnitude larger than that in the CV curve of ***t*** **-Bu-RuMOP**, which indicates that the presence of the conductive PANI near MOPs can also increase the electrochemically addressable amount of Ru­(III)/Ru­(II) sites in the MOPs. We thus further collected CV curves of thin films with **SO**_**3**_ **-RuMOP**-based nanocomposites. As revealed in Figure 8c, the first anodic peak of PANI located at +0.3 V shifts to around +0.39 V in the CV curve of the **PANI@SO**_**3**_ **-RuMOP** (1:1.5) thin film. The finding here suggests that, owing to the strong interaction between the **SO**_**3**_ **-RuMOP** and PANI in the nanocomposite, the redox reaction of MOPs, presumably coupled with the first redox reaction of PANI, could even shift the redox potential of the first set of CV peaks. CV data here indicate that MOPs incorporated within the nanocomposite not only act as the porous scaffold to support and confine the polyaniline but also provide additional Faradaic responses originating from their redox-active Ru­(III)/Ru­(II) clusters.

**Figure 8:**
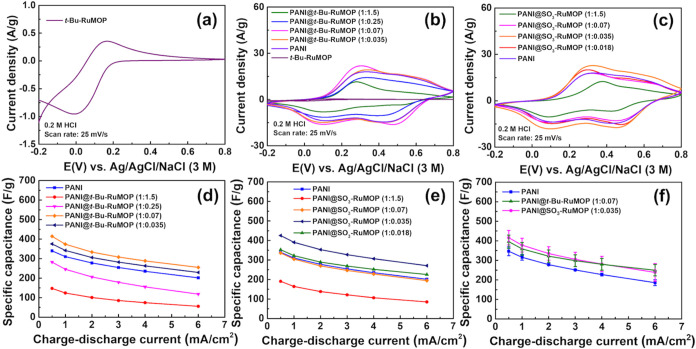
Cyclic voltammetry (CV) data of modified electrodes with (a) ***t*** **-Bu-RuMOP**, (b) ***t*** **-Bu-RuMOP**, PANI, and their nanocomposites, and (c) PANI and **SO**_**3**_ **-RuMOP**-based nanocomposites. All CV experiments were conducted in 0.2 M HCl (aq) at 25 mV/s. Values of specific capacitance calculated from GCD curves shown in Figures S15–S17 for (d) PANI and ***t*** **-Bu-RuMOP**-based nanocomposites and (e) PANI and **SO**_**3**_ **-RuMOP**-based nanocomposites. (f) Values of specific capacitance and the error bars collected from three separate modified electrodes.

To investigate the electrochemical kinetics of these nanocomposites, CV curves were measured at various scan rates (*v*), and the obtained data are plotted in Figures S13 and S14 for ***t*** **-Bu-RuMOP**-based nanocomposites and **SO**_**3**_ **-RuMOP**-based nanocomposites, respectively. From Figures S13 and S14, it can be observed that at fast scan rates, two sets of redox peaks in each CV curve tend to merge into one set of broad peaks. Values of cathodic peak current density (|*J*_pc_|) were then extracted from the most well-defined cathodic peak located between 0 V and +0.1 V in each CV curve, and the linear relationship between log­(|*J*_*p*c_|) and log­(*v*) was then obtained for each material; see the resulting slopes in Figures S13 and S14. In such plots, a slope of 0.5 suggests that the redox-active thin film exhibits a strongly diffusion-controlled characteristic during the electrochemical process.^52–54^ On the other hand, a slope of 1.0 in such plots indicates the similarity to monolayered redox-active sites, implying that all electrochemically addressable sites in the thin film could react at almost the same time.^22,52,53^ The slope of 1.0 in such plots can also come from the nonfaradaic process, if the nonfaradaic response is coupled within |*J*_pc_|.^52^ A slope closer to 1.0 is thus usually preferred for capacitive materials, as it indicates the presence of either a nonfaradaic process or redox reactions with facile kinetics. Herein, except for **PANI@SO**_**3**_ **-RuMOP** (1:1.5) and **PANI@SO**_**3**_ **-RuMOP** (1:0.018), all six other nanocomposites achieved slopes larger than 0.8, indicating the facile redox kinetics of these materials; these slopes are also quite similar to that of PANI reported previously.^22,23^ Another noticeable observation is that among all ***t*** **-Bu-RuMOP**-based nanocomposites, the **PANI@** ***t*** **-Bu-RuMOP** (1:0.07) shows the largest enclosed area in its CV curve as well as the largest slope in the plot of log­(|*J*_pc_|) vs log­(*v*) (Figure S13). On the other hand, for **SO**_**3**_ **-RuMOP**-based nanocomposites, as shown in Figure S14, **PANI@SO**_**3**_ **-RuMOP** (1:0.035) reveals the optimal performance. CV results here suggest that qualitatively these two nanocomposites should be optimal candidates for supercapacitors among their own series of materials.

To quantify the specific capacitance of each material, GCD experiments were conducted at various charge–discharge current densities (*J*), and the representative data are shown in Figures S15–S17. The specific capacitance (*C*) of each material was thereafter calculated by using the following equation^22,23^

1 $$C=\frac{J\times t_{d}}{V\times m}$$

where *t*_d_ is the discharging time from the GCD curve, *V* is the potential window, which is 1.0 V here, and *m* is the mass loading of the material, which is 0.173 mg/cm^2^ for all modified electrodes. The obtained values of specific capacitance for all materials are plotted in Figure 8d,e. For ***t*** **-Bu-RuMOP**-based nanocomposites, as shown in Figure 8d, their capacitance increases with the decreasing mass fraction of MOPs in the composite up to **PANI@** ***t*** **-Bu-RuMOP** (1:0.07), which possesses 9.2 wt % of MOPs in the material. By further decreasing the amount of MOPs, even though its performance is still better than that of the pristine PANI, the **PANI@** ***t*** **-Bu-RuMOP** (1:0.035) shows a worse capacitive performance compared to the **PANI@** ***t*** **-Bu-RuMOP** (1:0.07). For **SO**_**3**_ **-RuMOP**-based nanocomposites, a similar trend can be found in Figure 8e, with the **PANI@SO**_**3**_ **-RuMOP** (1:0.035), having 5.6 wt % of MOPs in the composite, as the optimal material for supercapacitors. At a charge–discharge rate of 0.5 mA/cm^2^, the **PANI@SO**_**3**_ **-RuMOP** (1:0.035) can achieve a specific capacitance of 416 ± 37 F/g, performing similarly compared to the **PANI@** ***t*** **-Bu-RuMOP** (1:0.07) (396 ± 34 F/g) and significantly outperforming the pristine PANI (347 ± 22 F/g). Findings here indicate that with a small mass fraction of MOPs (<10 wt %) in the nanocomposite to support the PANI as well as to provide the redox activity from their Ru_2_(II,III) paddlewheels, the resulting nanocomposites can exhibit much better capacitive performance compared to both the pristine MOPs and PANI. The electrostatic interaction between PANI and the discrete **SO**_**3**_ **-RuMOP** leads to optimal capacitive performance at a lower mass fraction of MOPs compared to nanocomposites based on aggregated ***t*** **-Bu-RuMOP**.

Values of specific capacitance of both optimal nanocomposites and the pristine PANI are plotted separately in Figure 8f for comparison, with error bars from three separate modified electrodes. Both nanocomposites with MOPs achieve comparable values of specific capacitance at each charge–discharge rate. However, both of them obviously outperform the pristine PANI, as the error bars for both composites in Figure 8f show almost no overlaps with those for the pristine PANI at most charge–discharge rates. In addition, slight differences in the rate capability can also be observed between these nanocomposites. For **PANI@SO**_**3**_ **-RuMOP** (1:0.035), it retains a specific capacitance of 238 ± 44 F/g at a fast charge–discharge rate of 6 mA/cm^2^, yielding a capacitance retention of 57% compared to the specific capacitance achieved at 0.5 mA/cm^2^. This capacitance retention is slightly lower than the 63% obtained for **PANI@** ***t*** **-Bu-RuMOP** (1:0.07). Compared to pristine PANI (54%), both nanocomposite materials exhibit higher capacitance retention. Findings here suggest that with the porous MOPs to support PANI and slightly hinder the aggregation of PANI during the polymerization, the resulting nanocomposite should provide a more facile mass transfer of counterions during the fast charge–discharge process; thus, this results in a better rate capability of the nanocomposite compared to the pristine PANI. The similar enhanced rate capability of PANI by porous supports was also observed in our previous work with MOF-PANI composites.^23^

Long-term GCD experiments were then conducted for 2000 cycles at a current density of 4 mA/cm^2^ with the use of **PANI@SO**_**3**_ **-RuMOP** (1:0.035), **PANI@** ***t*** **-Bu-RuMOP** (1:0.07), and the pristine PANI. As shown in Figure S18, the specific capacitance of **PANI@SO**_**3**_ **-RuMOP** (1:0.035) remained at 80% of its initial value after 2000 cycles of charge–discharge processes, outperforming the 72% retained by PANI and the 73% retained by **PANI@** ***t*** **-Bu-RuMOP** (1:0.07). This improved cycling stability of **PANI@SO**_**3**_ **-RuMOP** (1:0.035) is attributed to the homogeneous distribution of **SO**_**3**_ **-RuMOP** within PANI, which acts as a rigid scaffold and suppresses the swelling of PANI during long-term charging and discharging.^22^

## Conclusions

Two series of PANI@MOP nanocomposites with various PANI-to-MOP ratios were synthesized by conducting the *in situ* polymerization of aniline in the presence of ***t*** **-Bu-RuMOP** or **SO**_**3**_ **-RuMOP**, respectively. Both ***t*** **-Bu-RuMOP** and **SO**_**3**_ **-RuMOP** are negatively charged under the polymerization conditions, which could attract positively charged aniline monomers. This electrostatic interaction induces the polymerization occurring on the surface of dispersed MOPs or inside the MOPs. This allows for the synthesis of MOP-based composite particles with a rough polymer surface. DLS studies during the polymerization indicate that the ***t*** **-Bu-RuMOP**, which forms micrometer-sized aggregates in the solution, barely affects the hydrodynamic diameters of the generated PANI. On the other hand, **SO**_**3**_ **-RuMOP** are fully dispersed as molecular cages during the polymerization, which significantly reduces the hydrodynamic diameters of the product. Both ***t*** **-Bu-RuMOP** and **SO**_**3**_ **-RuMOP** contribute to pseudocapacitance in the corresponding PANI@MOP composites. Composites with the optimal capacitive performance in both series, **PANI@SO**_**3**_ **-RuMOP** (1:0.035) and **PANI@** ***t*** **-Bu-RuMOP** (1:0.07), achieve specific capacitances of 416 ± 37 F/g and 396 ± 34 F/g at 0.5 mA/cm^2^, respectively, which significantly outperform the pristine PANI (347 ± 22 F/g). In addition, both the **PANI@SO**_**3**_ **-RuMOP** (1:0.035) and **PANI@** ***t*** **-Bu-RuMOP** (1:0.07) exhibit a higher rate capability and better long-term cycling stability compared to the pristine PANI. Findings here suggest that a small amount of redox-active RuMOP as the additive during the polymerization, i.e., 5.6 wt % here for **PANI@SO**_**3**_ **-RuMOP** (1:0.035), enhances the electrochemical performance of PANI. As a result, the nanocomposite exhibits higher specific capacitance, rate capability, and long-term capacitance retention compared with pristine PANI.

## Supplementary Material



## Notes

Experimental details, DLS data, additional SEM and TEM data, ICP-OES results, N_2_ sorption isotherms, and additional electrochemical data. (PDF)
