# Supplementary material for: In Situ Polymerized Polyaniline in Redox-Active Metal–Organic Polyhedra for Supercapacitors
Source: ACS Appl Mater Interfaces. 2026 Jun 12;18(25):35348–59. doi: 10.1021/acsami.6c06826 (PMC13339017; doi:10.1021/acsami.6c06826)
Supplement: Supplementary file 1 [file am6c06826_si_001.pdf]

Supporting Information

In Situ-Polymerized Polyaniline in Redox-Active Metal–Organic Polyhedra for Supercapacitors

Yan-Ling Chang,[a,†] Fuerkaiti Tayier,[b,c,†] Cheng-Yan Hsieh,[a] Shuhei Furukawa,[b,c,*] and Chung-Wei Kung [a,d,*]

[a] Department of Chemical Engineering, National Cheng Kung University, Tainan City, 70101, Taiwan.

[b] Institute for Integrated Cell-Material Sciences (WPI-iCeMS), Kyoto University, Yoshida, Sakyo-ku, Kyoto 606-8501, Japan.

[c] Department of Synthetic Chemistry and Biological Chemistry, Graduate School of Engineering, Kyoto University, Katsura, Nishikyo-ku, Kyoto 615-8510, Japan.

[d] Program on Key Materials, Academy of Innovative Semiconductor and Sustainable Manufacturing, National Cheng Kung University, Tainan City, 70101, Taiwan.

[†] These authors contributed equally: Yan-Ling Chang, Fuerkaiti Tayier.

* Corresponding authors:

shuhei.furukawa@icems.kyoto-u.ac.jp (S. F.); cwkung@mail.ncku.edu.tw (C.-W. K.)

Synthesis of [$Ru_2(OAc)_4(THF)_2$](BF_4)

The starting cationic diruthenium complex, [$Ru_2(OAc)_4(THF)_2$](BF_4) (THF = tetrahydrofuran) was prepared by following a previously reported method.[1] 115 mM $RuCl_3$ and 5 equivalents of LiCl were added to a 1:5 mixture of acetic anhydride and acetic acid. The brown mixture was saturated with O_2, then refluxed overnight to obtain a reddish-brown suspension. This reddish-brown suspension was centrifuged to obtain the $Ru_2(OAc)_4Cl$ as a solid precipitate. $Ru_2(OAc)_4Cl$ was washed with acetic acid. After washing, $Ru_2(OAc)_4Cl$ was suspended in MeOH and then collected with Et_2O. 50 mM $Ru_2(OAc)_4Cl$ and 1 equivalent of $AgBF_4$ were added into THF to synthesize [$Ru_2(OAc)_4(THF)_2$](BF_4). After stirring overnight at RT, a white precipitate and reddish-brown solution were obtained. The solvent was removed from the reddish-brown solution using a rotavapor, yielding the reddish-brown solid [$Ru_2(OAc)_4(THF)_2$](BF_4).

Synthesis of [Ru_2(*t*-Bu-bdc)$_2$]$_{12}$(BF_4)$_{12}$ (*t*-Bu-RuMOP, *t*-Bu-bdc = 5-*tert*-butylbenzene-1,3-dicarboxylate)

***t*-Bu-RuMOP** was synthesized following a previously reported protocol.[2] [$Ru_2(OAc)_4(THF)_2$](BF_4) (0.180 g, 0.27 mmol) and 5-*tert*-butylbenzene-1,3-dicarboxylic acid (0.230 g, 1.03 mmol) were dissolved in 1 mL of DMA. Na_2CO_3 (0.020 g, 0.19 mmol) was added to the solution. The mixture was sealed in a vial and then heated at 120 °C for 16 h. Brown crystals of ***t*-Bu-RuMOP** were obtained. The crystals were washed with DMA, water, and methanol. The solid was collected by centrifugation. The collected solid was suspended in approximately 4 mL of methanol. Aqueous HBF_4 (50 wt.%) was added to the suspension. The mixture was centrifuged, and the precipitates were collected. The obtained yellowish-brown solids were dissolved in DMF. The DMF solution was poured into diethyl ether to induce precipitation. The precipitate was collected by centrifugation, washed with diethyl ether, and dried under ambient conditions. The resulting powder dissolves in various organic solvents and was used for all subsequent experiments.

Synthesis of $[Ru_2(SO_3\text{-bdc})(HSO_3\text{-bdc})]_{12}$ (SO_3-RuMOP, SO_3-bdc = 5-sulfonate-1,3-benzenedicarboxylate, HSO_3-bdc = 5-sulfonic acid-1,3-benzenedicarboxylate)

SO_3-RuMOP was synthesized according to previous reports with modifications.[2-3] $[Ru_2(OAc)_4(THF)_2](BF_4)$ (0.200 g, 0.30 mmol) and sodium 5-sulfonate-1,3-benzenedicarboxylic acid (0.200 g, 0.75 mmol) were dissolved in 4 mL of a DMA:water (9:1) mixture. The solution was sealed in a vial and heated at 120 °C for 16 h. Red-brown crystals of **SO_3-RuMOP** formed after heating. The crystals were washed with DMA and acetone. The solid was collected by centrifugation. The collected solid was suspended in approximately 10 mL of MeOH. Triflic acid (1 mL) was added to the suspension. The resulting mixture was poured into 30 mL of diethyl ether. This acid treatment and ether precipitation procedure was repeated three times. The final suspension was centrifuged to obtain a brown powder. The powder was dried under ambient conditions and used for all experiments.

Synthesis of PANI@SO_3-RuMOP (1:X) and PANI@*t*-Bu-RuMOP (1:X) (PANI = polyaniline)

To polymerize aniline in the presence of MOPs, similar procedures of *in situ* polymerization for MOFs reported in our previous work were employed.[4-5] The obtained nanocomposites with **SO_3-RuMOP** and ***t*-Bu-RuMOP** were named as "**PANI@SO_3-RuMOP** (1:*X*)" and "**PANI@*t*-Bu-RuMOP** (1:*X*)," respectively, where *X* corresponds to the mass ratio between the MOP and aniline added during the synthesis. First, 14 μL of aniline was dissolved in a solution containing 3.75 mL of deionized water (H_2O) and 0.155 mL of concentrated HCl. The solution was stirred at 1000 rpm for 30 min and used as a solvent. Aqueous suspensions of **SO_3-RuMOP** and ***t*-Bu-RuMOP** were prepared separately by sonication. Each suspension contains 3.75 mL of solvent and a solid concentration of 5.60, 0.93, 0.27, 0.13, or 0.067 mg/mL. These concentrations correspond to *X* values of 1.5, 0.25, 0.07, 0.035, or 0.018, respectively. The RuMOP suspension was then added to the aniline solution. The mixture was stirred at 1000 rpm for 6 h to allow the uniform penetration of aniline monomers into

MOPs. In the meantime, 40 mg of APS was dissolved in 2.5 mL of H_2O. The APS solution was added to the mixture containing MOP and aniline under continuous stirring, with an injection rate of 0.25 mL/min. The mixture was stirred at 1000 rpm overnight to complete the polymerization. The obtained solid was washed with 10 mL of 0.2 M HCl aqueous solution three times through centrifugation. After removal of the aqueous supernatant, 10 mL of acetone was added to wash the solid, and centrifugation was performed again to remove the supernatant. Then, the solid was washed three times with 10 mL of diethyl ether. The immersion periods were 2 h, overnight, and 2 h. After removing the final supernatant, the solid was dried under vacuum at room temperature overnight to obtain the final product.

Synthesis of pristine PANI

To synthesize the pristine PANI for comparison, the same procedure was used except that the 3.75 mL of MOP suspension was replaced by 3.75 mL of H_2O.

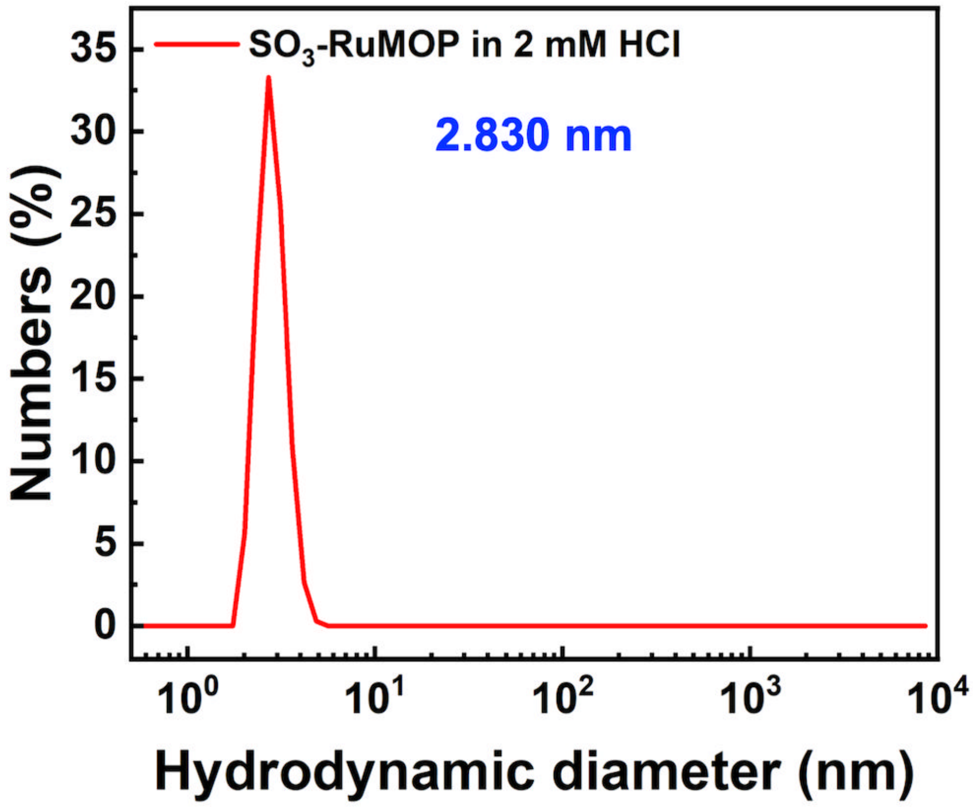

Figure S1. DLS data of **SO$_3$-RuMOP** in an aqueous solution containing 2 mM of HCl.

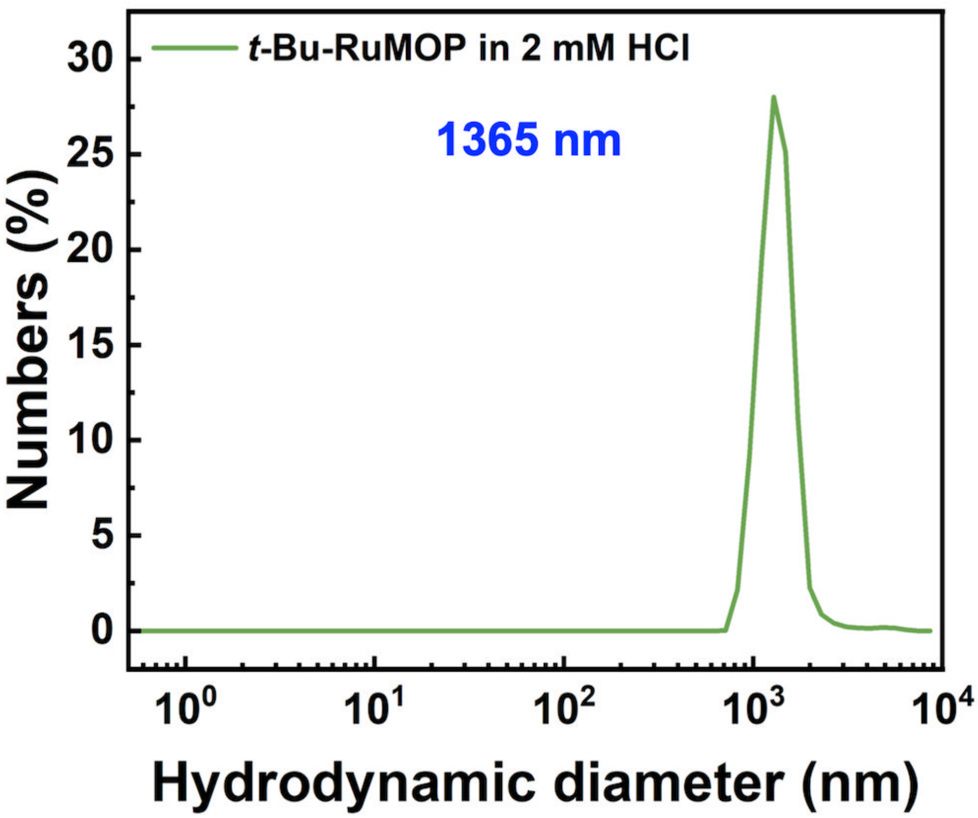

Figure S2. DLS data of ***t*-Bu-RuMOP** suspension. The 1 mg of ***t*-Bu-RuMOP** was suspended in 1 mL of aqueous solution containing 2 mM HCl. The suspension was kept for 10 min to allow large particles to precipitate, and the stable supernatant was used for measurement.

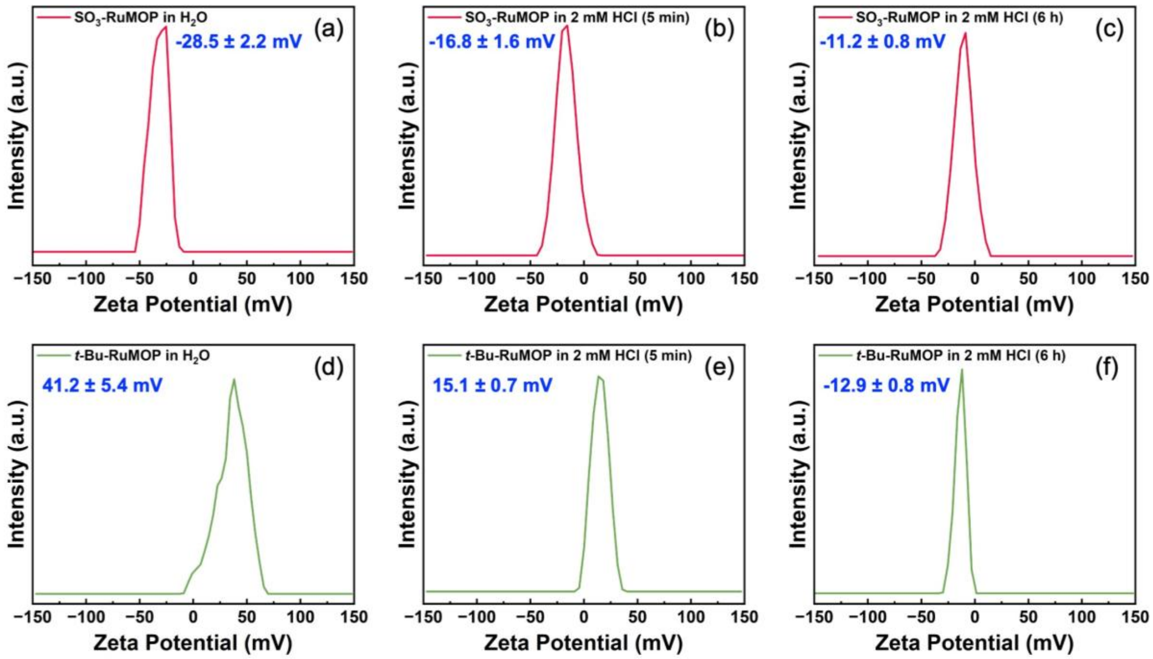

Figure S3. Zeta potential of **SO_3-RuMOP** in (a) H_2O, (b) aqueous HCl, and (c) aqueous HCl after 6 h; red lines. Zeta potential of ***t*-Bu-RuMOP** in (d) H_2O, (e) aqueous HCl, and (f) aqueous HCl after 6 h; green lines. RuMOPs were stirred in 200 mM aqueous HCl, which corresponds to the conditions used for complex synthesis. Before zeta potential measurements, the samples were diluted to 2 mM HCl to maintain a low ionic strength suitable for zeta potential measurements.

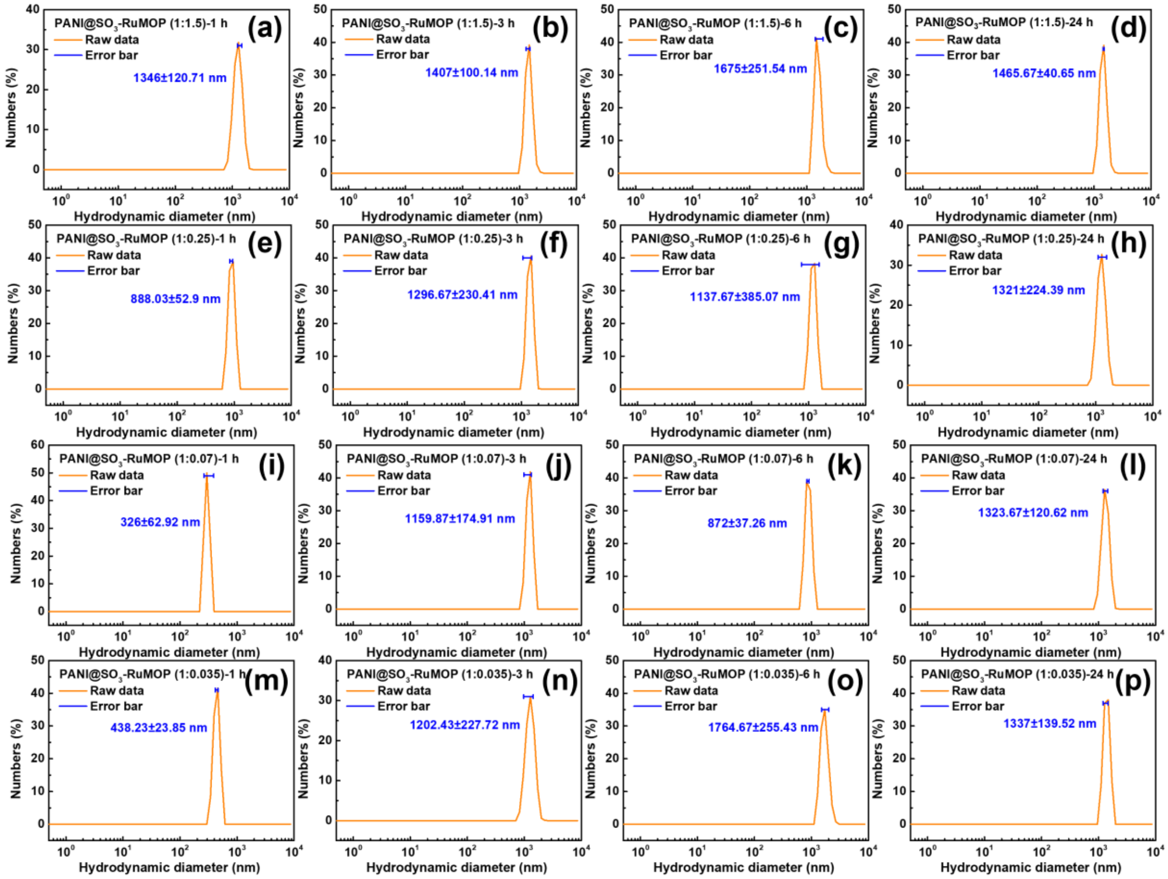

Figure S4. Representative DLS data of suspensions collected during the synthesis of (a-d) **PANI@SO3-RuMOP** (1:1.5), (e-h) **PANI@SO3-RuMOP** (1:0.25), (i-l) **PANI@SO3-RuMOP** (1:0.07), and (m-p) **PANI@SO3-RuMOP** (1:0.035), collected at 1, 3, 6, and 24 h after adding APS, respectively.

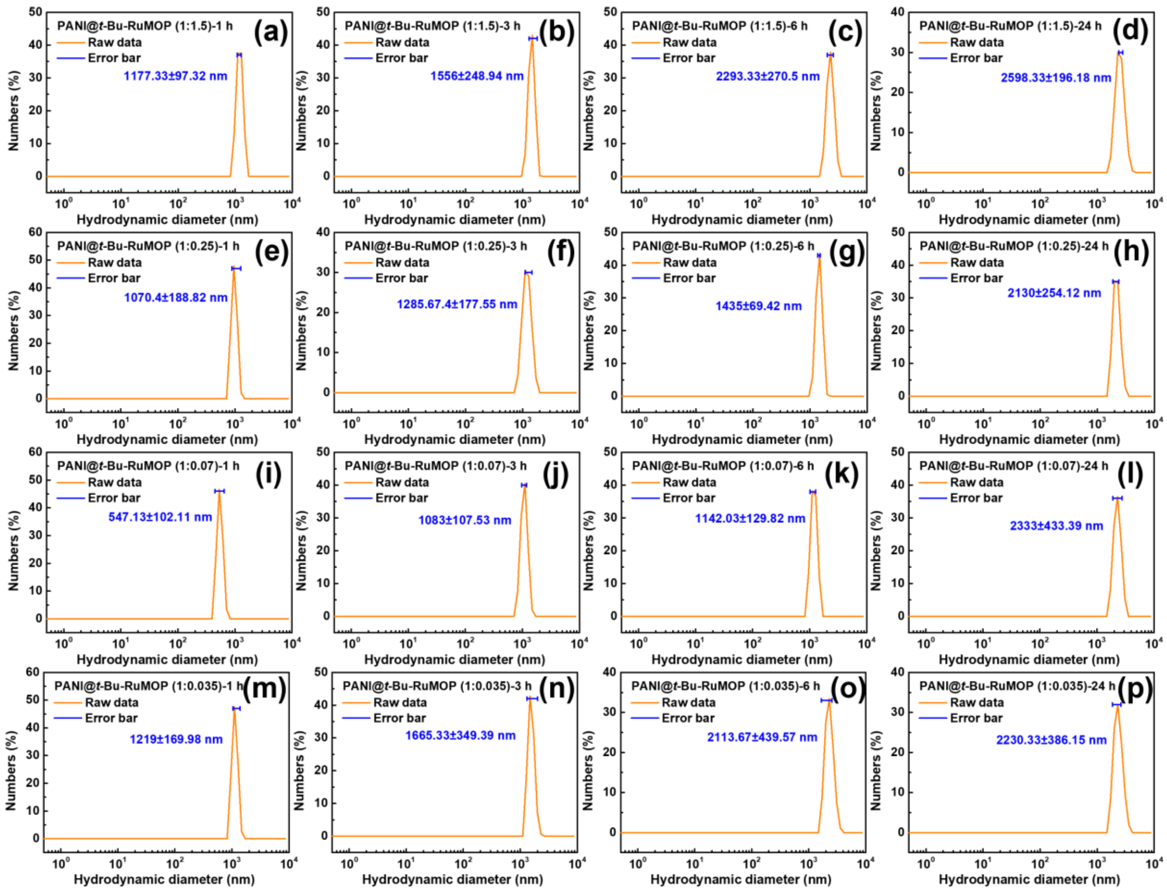

Figure S5. Representative DLS data of suspensions collected during the synthesis of (a-d) **PANI@*t*-Bu-RuMOP** (1:1.5), (e-h) **PANI@*t*-Bu-RuMOP** (1:0.25), (i-l) **PANI@*t*-Bu-RuMOP** (1:0.07), and (m-p) **PANI@*t*-Bu-RuMOP** (1:0.035), collected at 1, 3, 6, and 24 h after adding APS, respectively.

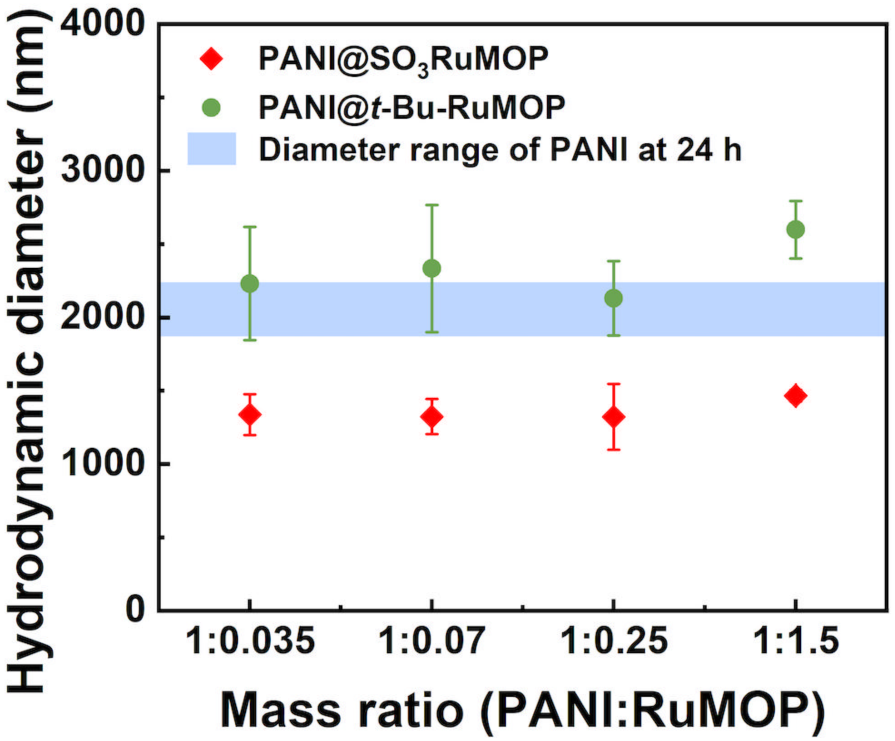

Figure S6. DLS measurements of **PANI@SO3-RuMOP** (1:*X*) and **PANI@*t*-Bu-RuMOP** (1:*X*) (*X* = 1.5, 0.25, 0.07, 0.035) recorded at 24 h. The blue area represents the size range of PANI at 24 h. Error bars are taken from the standard deviation of three independent experiments. Red, **PANI@SO3-RuMOP** (1:*X*) at 24 h; green, **PANI@*t*-Bu-RuMOP** (1:*X*) at 24 h.

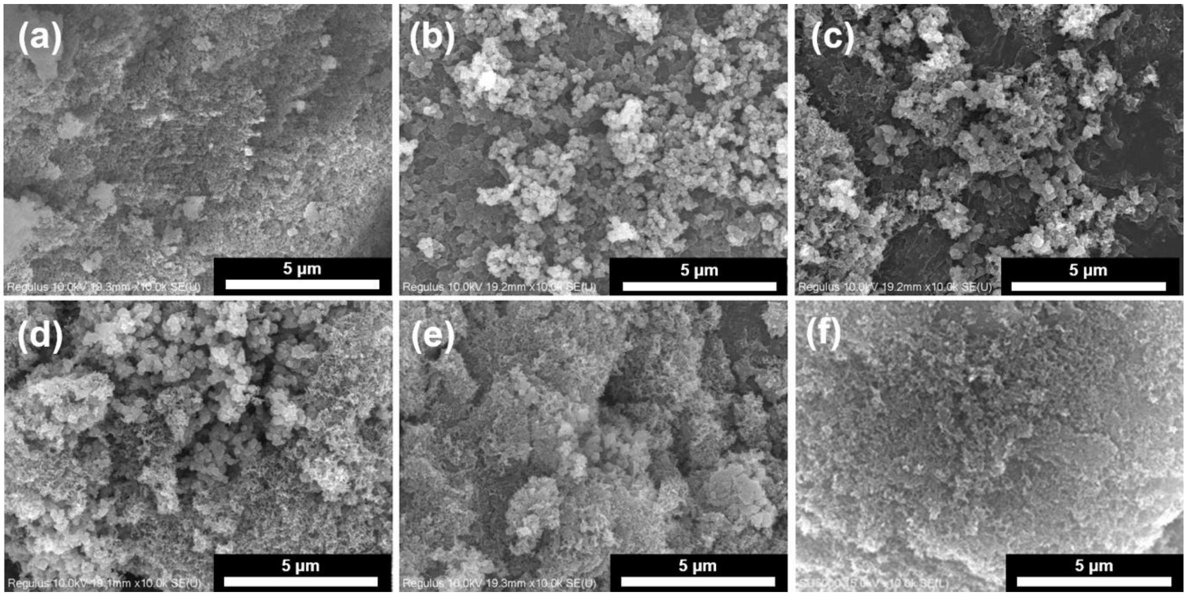

Figure S7. Low-magnification SEM images of (a) **SO_3-RuMOP**, (b) **PANI@SO_3-RuMOP** (1:1.5), (c) **PANI@SO_3-RuMOP** (1:0.07), (d) **PANI@SO_3-RuMOP** (1:0.035), (e) **PANI@SO_3-RuMOP** (1:0.018), and (f) PANI.

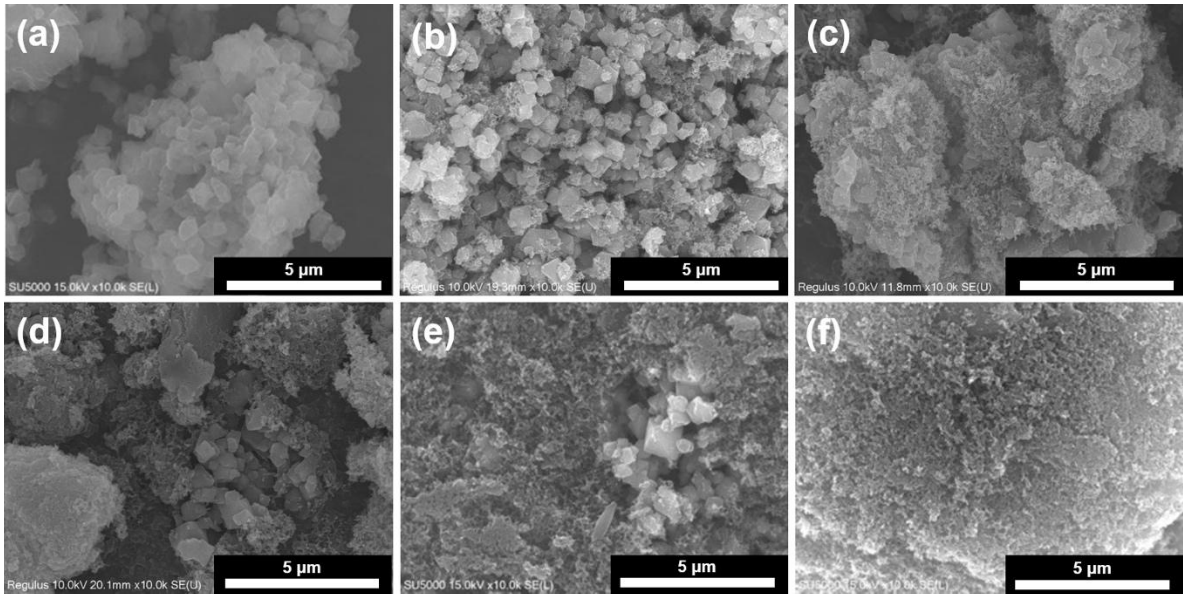

Figure S8. Low-magnification SEM images of (a) ***t*-Bu-RuMOP**, (b) **PANI@*t*-Bu-RuMOP** (1:1.5), (c) **PANI@*t*-Bu-RuMOP** (1:0.25), (d) PANI@*t*-Bu-RuMOP (1:0.07), (e) **PANI@*t*-Bu-RuMOP** (1:0.035), and (f) PANI.

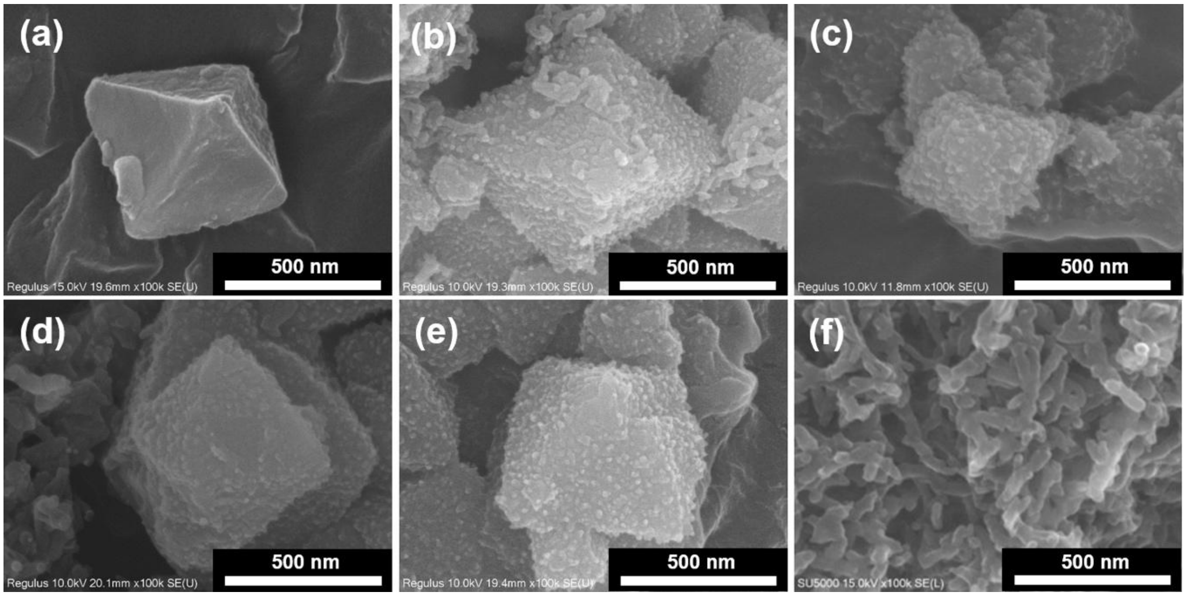

Figure S9. SEM images of (a) ***t*-Bu-RuMOP**, (b) **PANI@*t*-Bu-RuMOP** (1:1.5), (c) **PANI@*t*-Bu-RuMOP** (1:0.25), (d) **PANI@*t*-Bu-RuMOP** (1:0.07), (e) **PANI@*t*-Bu-RuMOP** (1:0.035), and (f) PANI, collected at a high magnification.

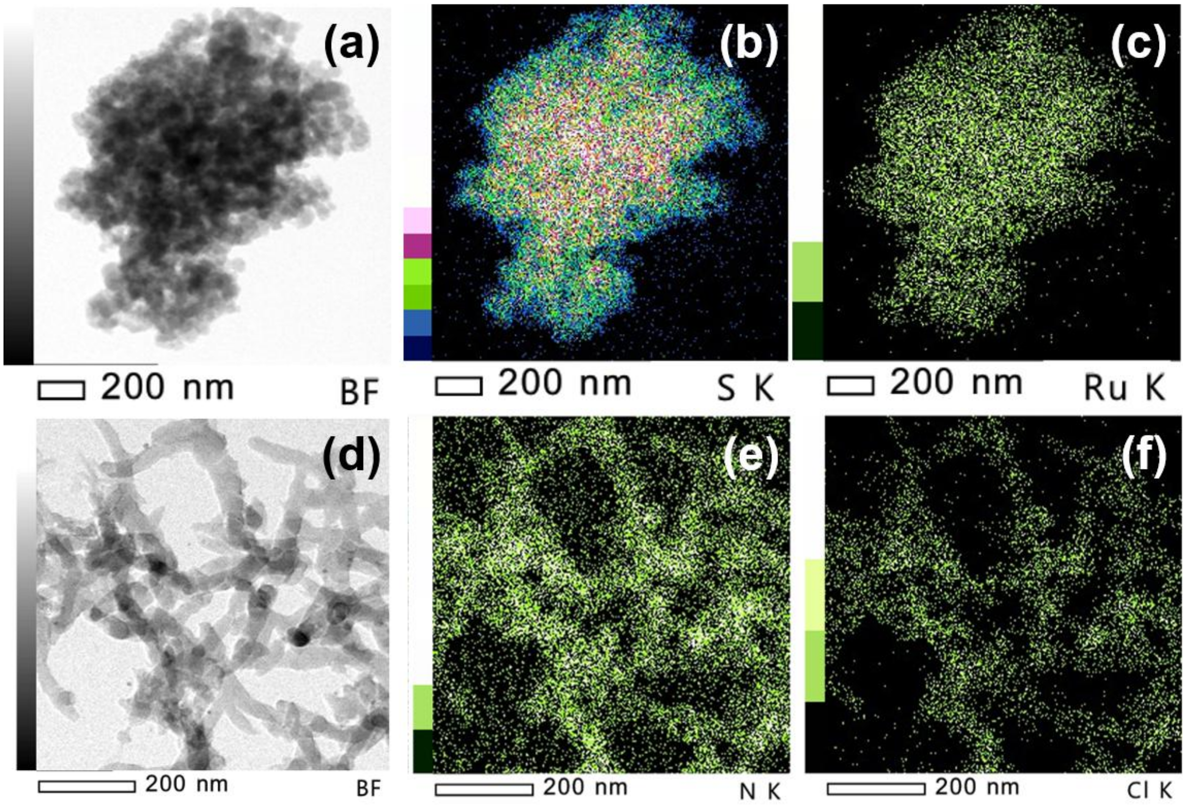

Figure S10. TEM images of (a) **SO_3-RuMOP** and (d) PANI. EDS mapping signals of (b) sulfur and (c) ruthenium, collected from the region shown in (a). EDS mapping signals of (e) nitrogen and (f) chlorine, collected from the region shown in (d).

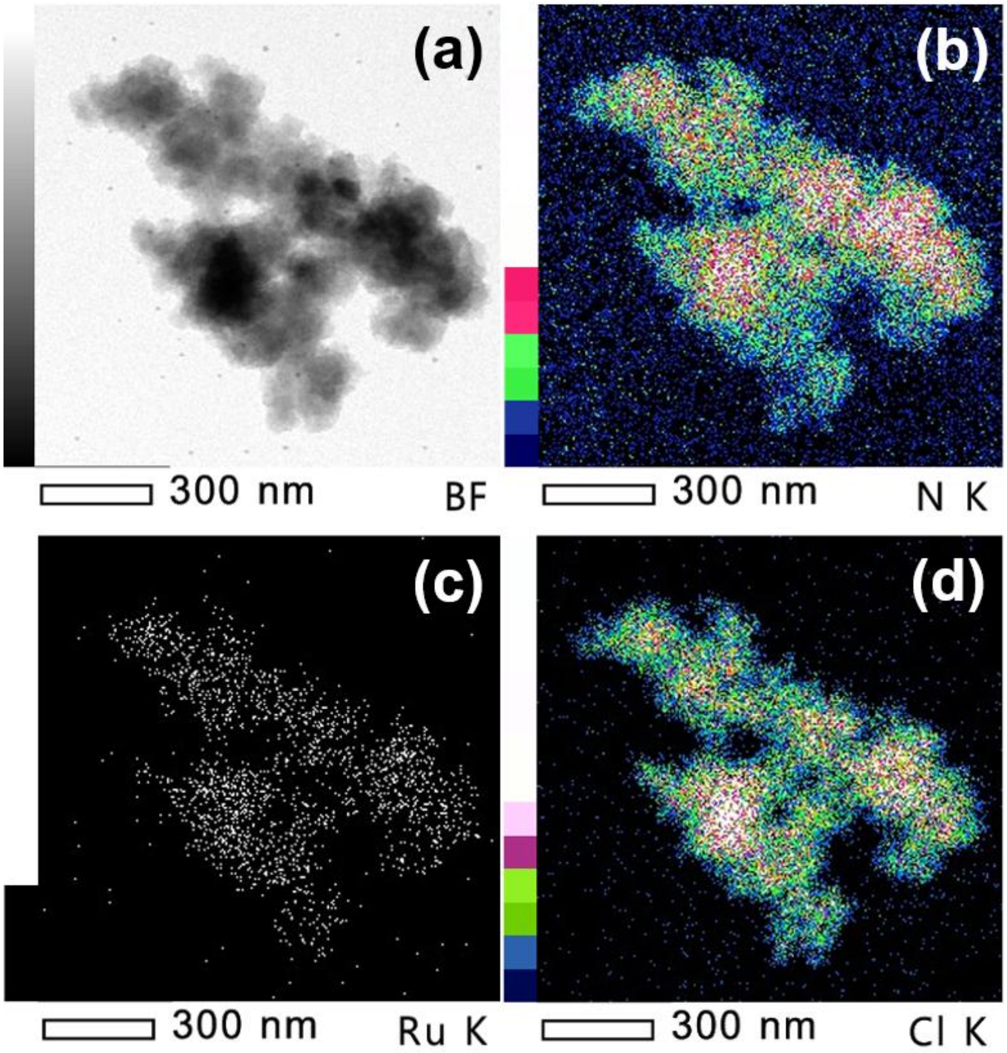

Figure S11. (a) TEM image of **PANI@SO_3-RuMOP** (1:0.035). EDS mapping signals of (b) nitrogen, (c) ruthenium, and (d) chloride, collected from the region shown in (a).

Table S1. ICP-OES results for **SO_3-RuMOP**-based materials. Accurately weighted 3.0 mg of each material was digested, followed by the dilution to 40 mL to form the ICP-OES sample.

Material	Ru in ICP sample (mg/L)	Ru in ICP sample (μmol)	MOPs (wt%)
SO_3-RuMOP	19.48	7.71	100.0
PANI@SO_3-RuMOP (1:1.5)	13.03	5.16	66.9
PANI@SO_3-RuMOP (1:0.07)	2.11	0.84	10.8
PANI@SO_3-RuMOP (1:0.035)	1.09	0.43	5.6
PANI@SO_3-RuMOP (1:0.018)	0.53	0.21	2.7

Table S2. ICP-OES results for ***t*-Bu-RuMOP**-based materials. Accurately weighted 3.0 mg of each material was digested, followed by the dilution to 40 mL to form the ICP-OES sample.

Material	Ru in ICP sample (mg/L)	Ru in ICP sample (μmol)	MOPs (wt%)
t-Bu-RuMOP	20.90	8.27	100.0
PANI@*t*-Bu-RuMOP (1:1.5)	14.88	5.89	71.2
PANI@*t*-Bu-RuMOP (1:0.25)	4.81	1.90	23.0
PANI@*t*-Bu-RuMOP (1:0.07)	1.93	0.76	9.2
PANI@*t*-Bu-RuMOP (1:0.035)	0.71	0.28	3.4

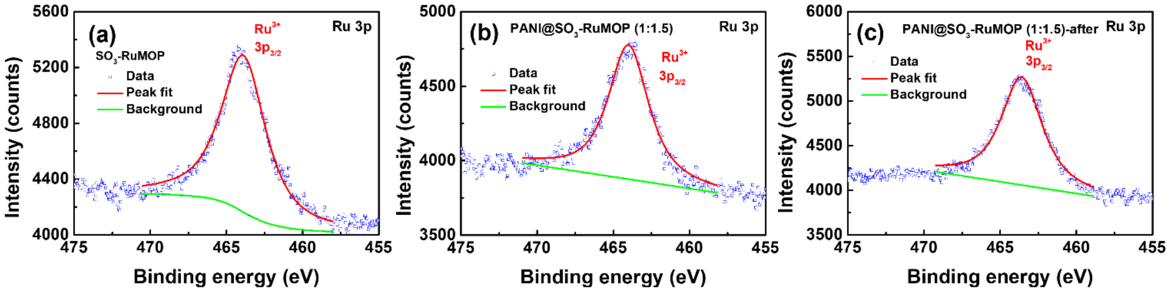

Figure S12. XPS spectra of (a) **SO_3-RuMOP**, (b) **PANI@SO_3-RuMOP** (1:1.5), and (c) thin film of **PANI@SO_3-RuMOP** (1:1.5) after 20 cycles of CV scans, collected in the region of Ru 3p.

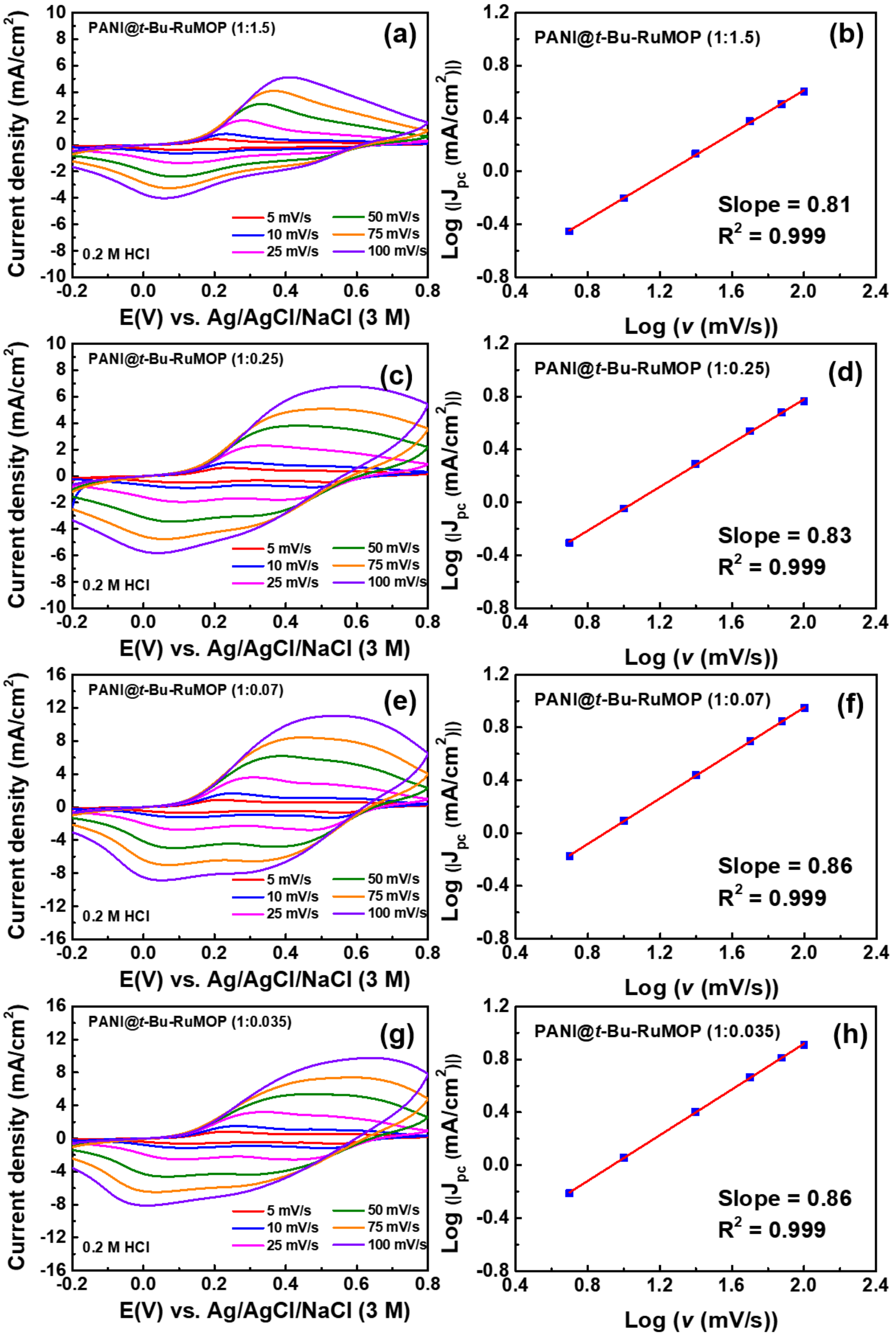

Figure S13. CV curves of modified electrodes with (a) **PANI@*t*-Bu-RuMOP** (1:1.5), (c) **PANI@*t*-Bu-RuMOP** (1:0.25), (e) **PANI@*t*-Bu-RuMOP** (1:0.07), and (g) **PANI@*t*-Bu-RuMOP** (1:0.035), collected at various scan rates (v). Plots of log($|J_{pc}|$) *vs.* log(v) extracted from (a), (c), (e), and (g) are shown in (b), (d), (f), and (h), respectively. Aqueous solutions containing 0.2 M of HCl were used as electrolytes for all CV experiments.

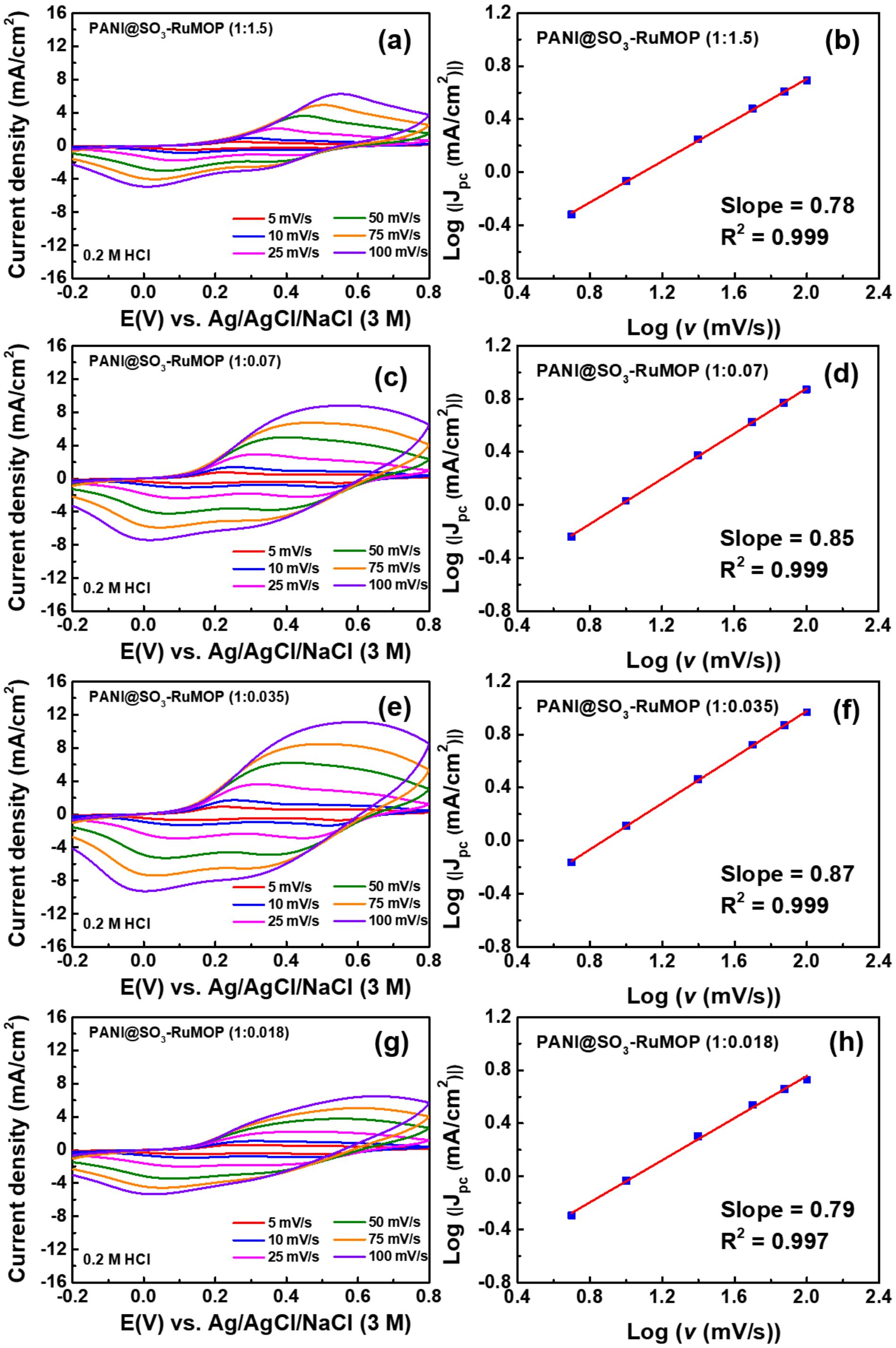

Figure S14. CV curves of (a) **PANI@SO3-RuMOP** (1:1.5), (c) **PANI@SO3-RuMOP** (1:0.07), (e) **PANI@SO3-RuMOP** (1:0.035), and (g) **PANI@SO3-RuMOP** (1:0.018), collected at various scan rates (v). Plots of log(|J_{pc}|) *vs.* log(v) extracted from (a), (c), (e), and (g) are shown in (b), (d), (f), and (h), respectively. Aqueous solutions containing 0.2 M of HCl were used as electrolytes for all CV experiments.

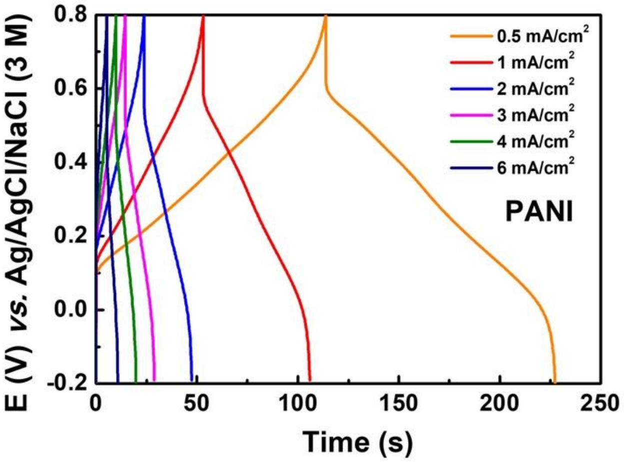

Figure S15. GCD curves of the modified electrode with PANI, measured in an aqueous solution containing 0.2 M of HCl.

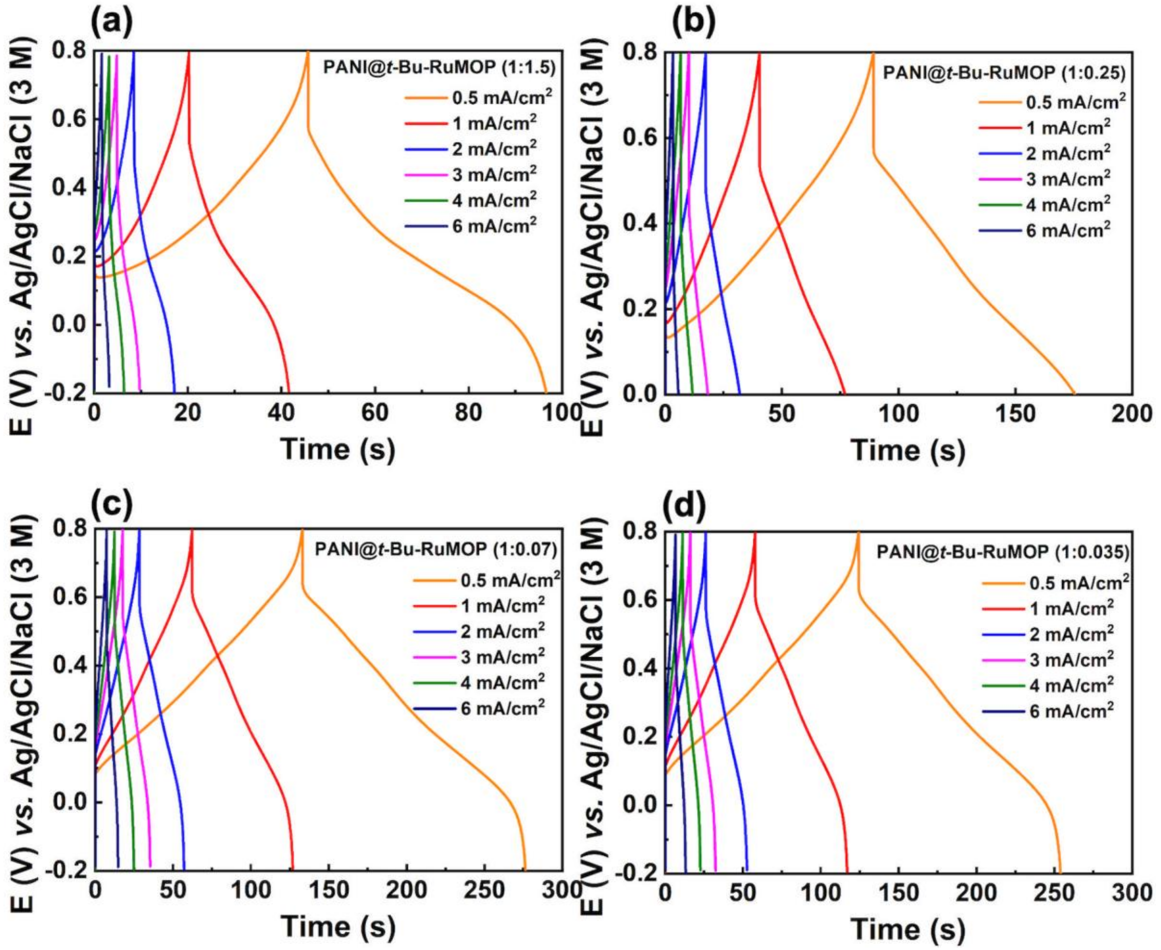

Figure S16. GCD curves of modified electrodes with (a) **PANI@*t*-Bu-RuMOP** (1:1.5), (b) **PANI@*t*-Bu-RuMOP** (1:0.25), (c) **PANI@*t*-Bu-RuMOP** (1:0.07), and (d) **PANI@*t*-Bu-RuMOP** (1:0.035). Aqueous solutions containing 0.2 M of HCl were used as electrolytes for all GCD experiments.

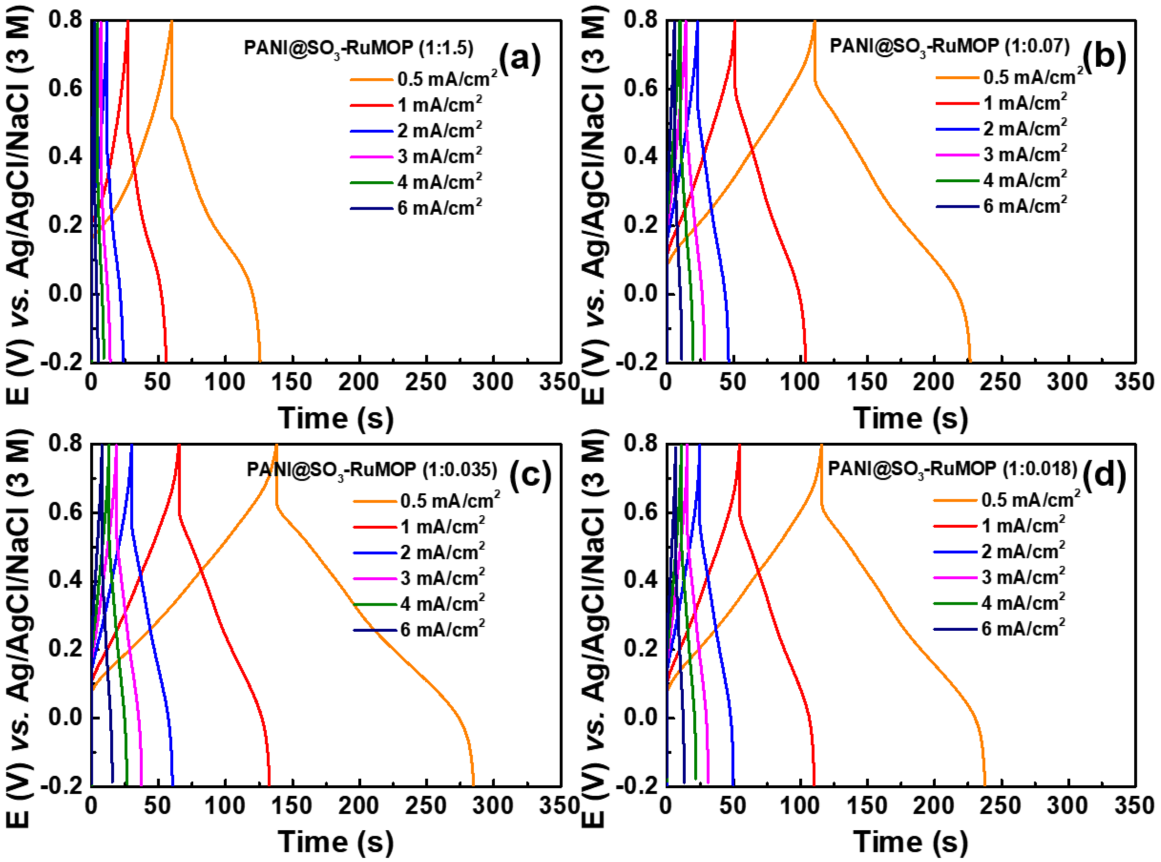

Figure S17. GCD curves of modified electrodes with (a) **PANI@SO_3-RuMOP** (1:1.5), (b) **PANI@SO_3-RuMOP** (1:0.07), (c) **PANI@SO_3-RuMOP** (1:0.035), and (d) **PANI@SO_3-RuMOP** (1:0.018). Aqueous solutions containing 0.2 M of HCl were used as electrolytes for all GCD experiments.

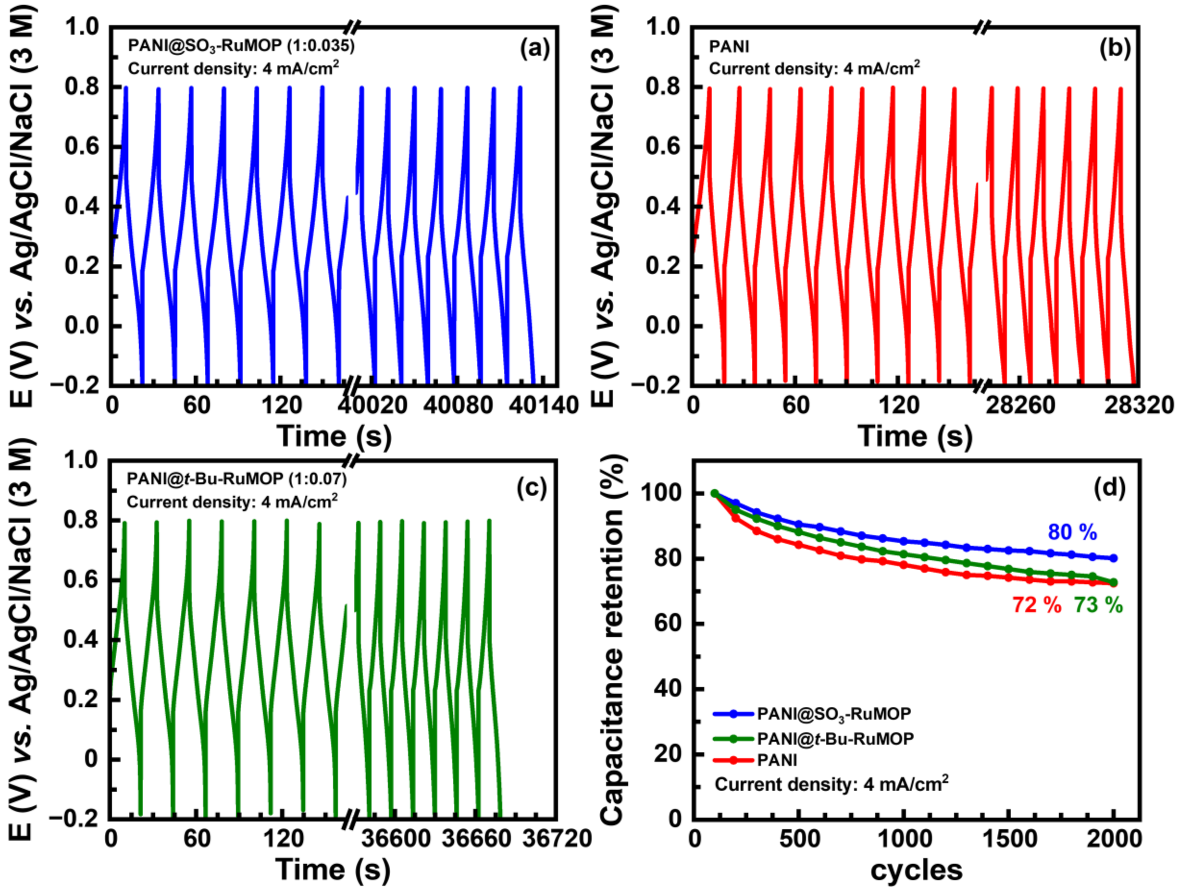

Figure S18. GCD curves of modified electrodes with (a) **PANI@SO3-RuMOP** (1:0.035), (b) PANI, and (c) **PANI@*t*-Bu-RuMOP** (1:0.07) measured at a charge-discharge current density of 4 mA/cm^2 for 2000 cycles. Aqueous solutions containing 0.2 M of HCl were used as electrolytes for all GCD experiments. (d) Capacitance retentions of **PANI@SO3-RuMOP** (1:0.035), PANI, and **PANI@*t*-Bu-RuMOP** (1:0.07) obtained from (a), (b), and (c), respectively.

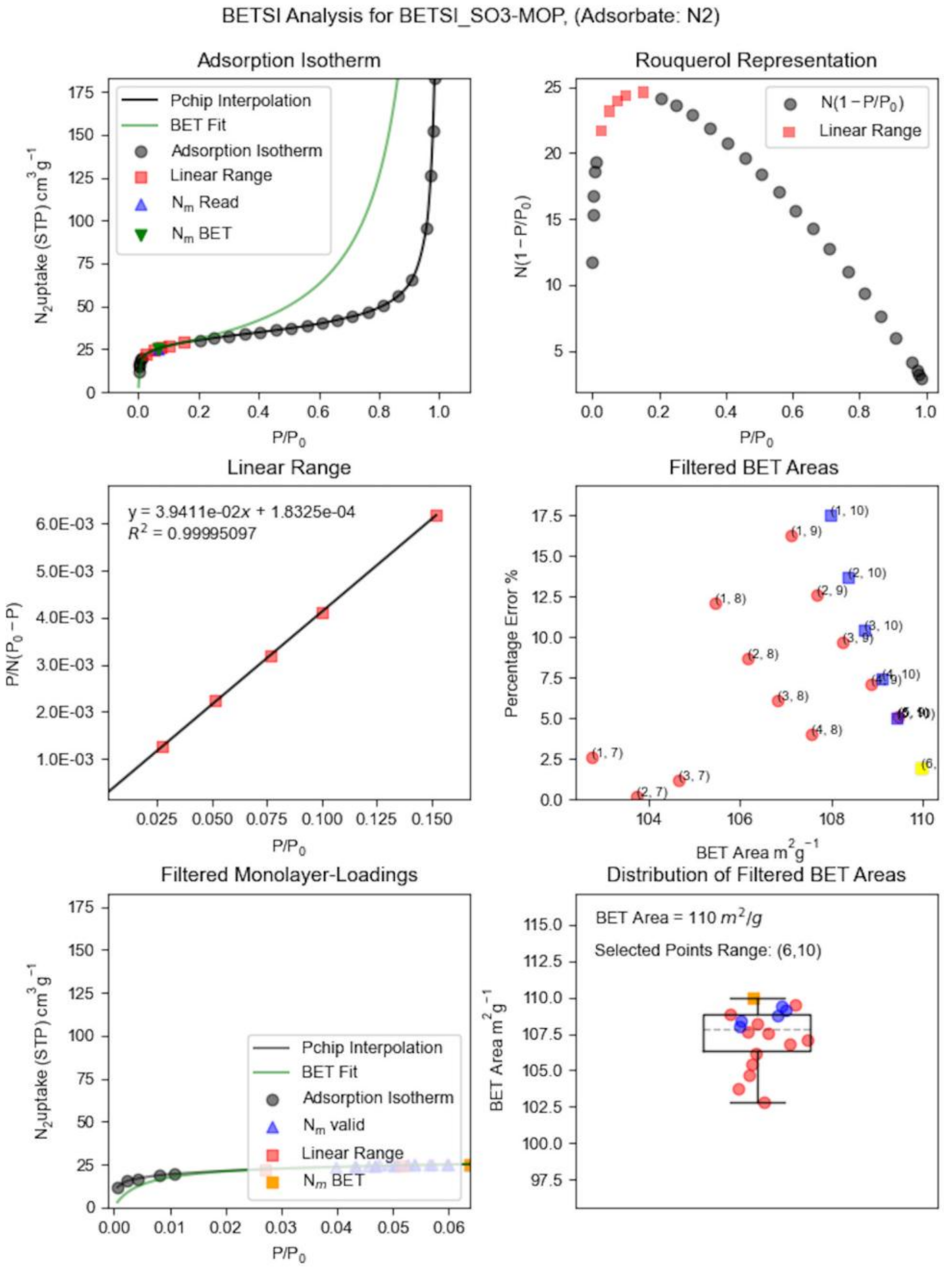

Figure S19. BETSI analysis of **SO_3-RuMOP**, generated by the BETSI program.[6]

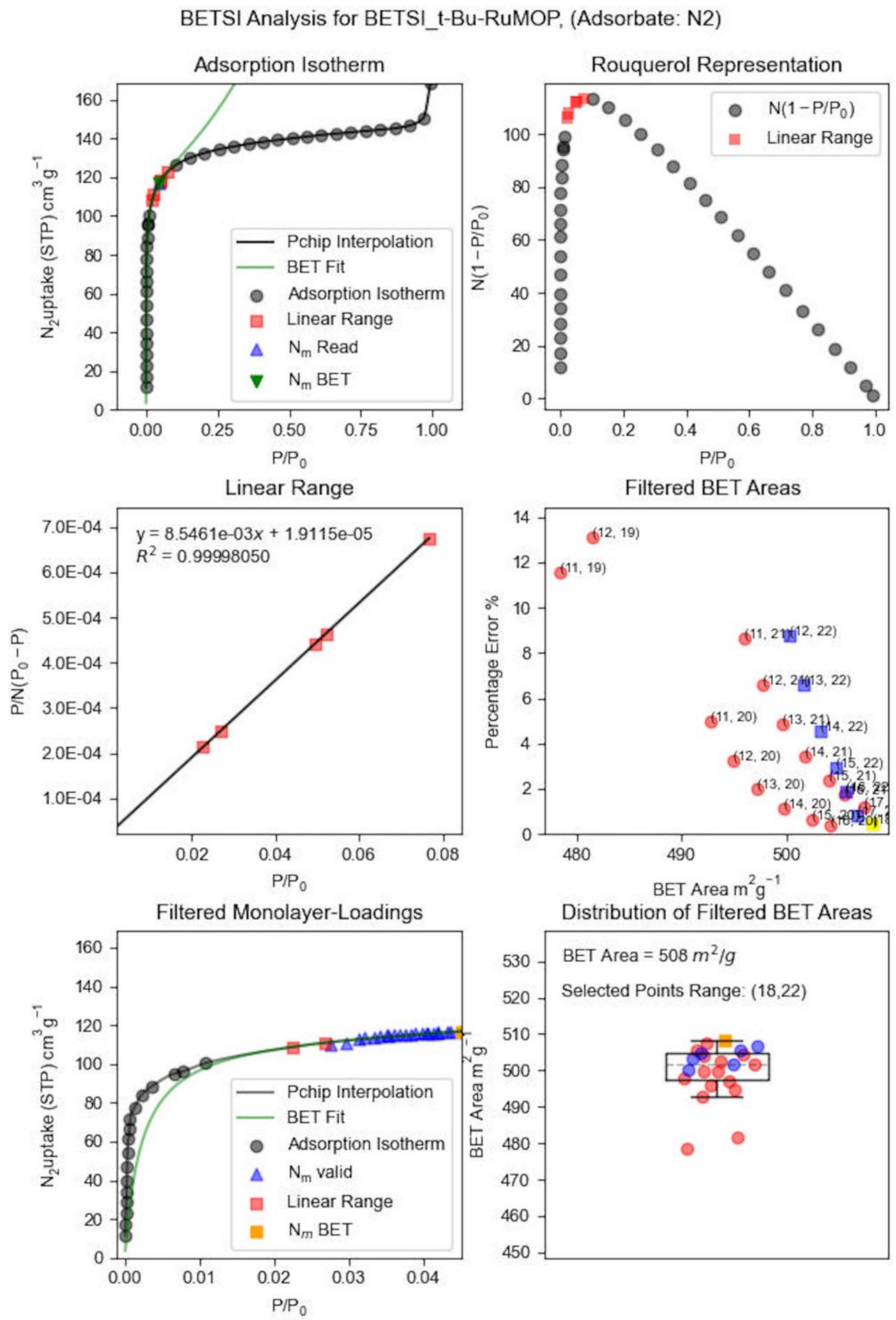

Figure S20. BETSI analysis of ***t*-Bu-RuMOP**, generated by the BETSI program.[6]

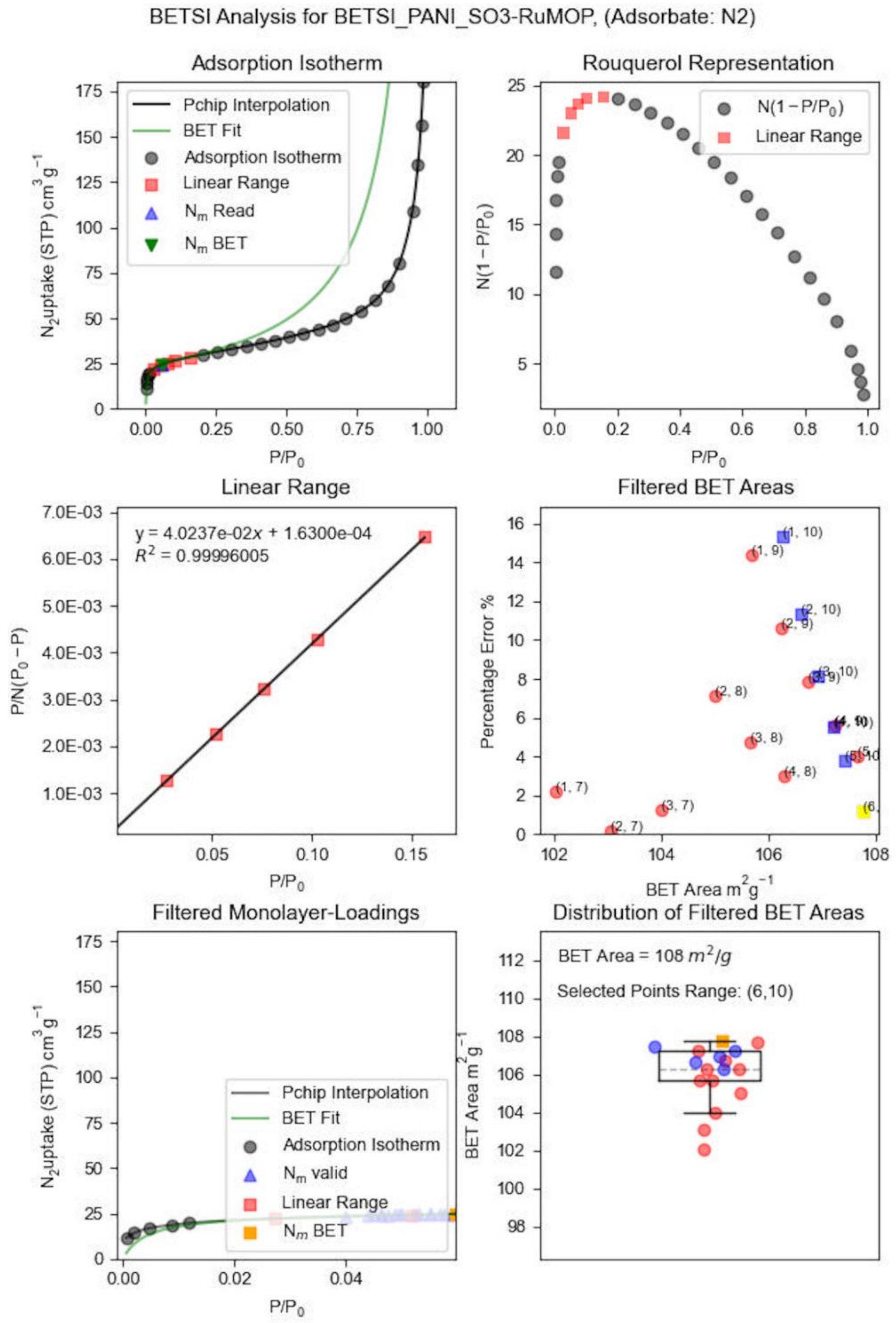

Figure S21. BETSI analysis of **PANI@SO₃-RuMOP** (1:1.5), generated by the BETSI program.[6]

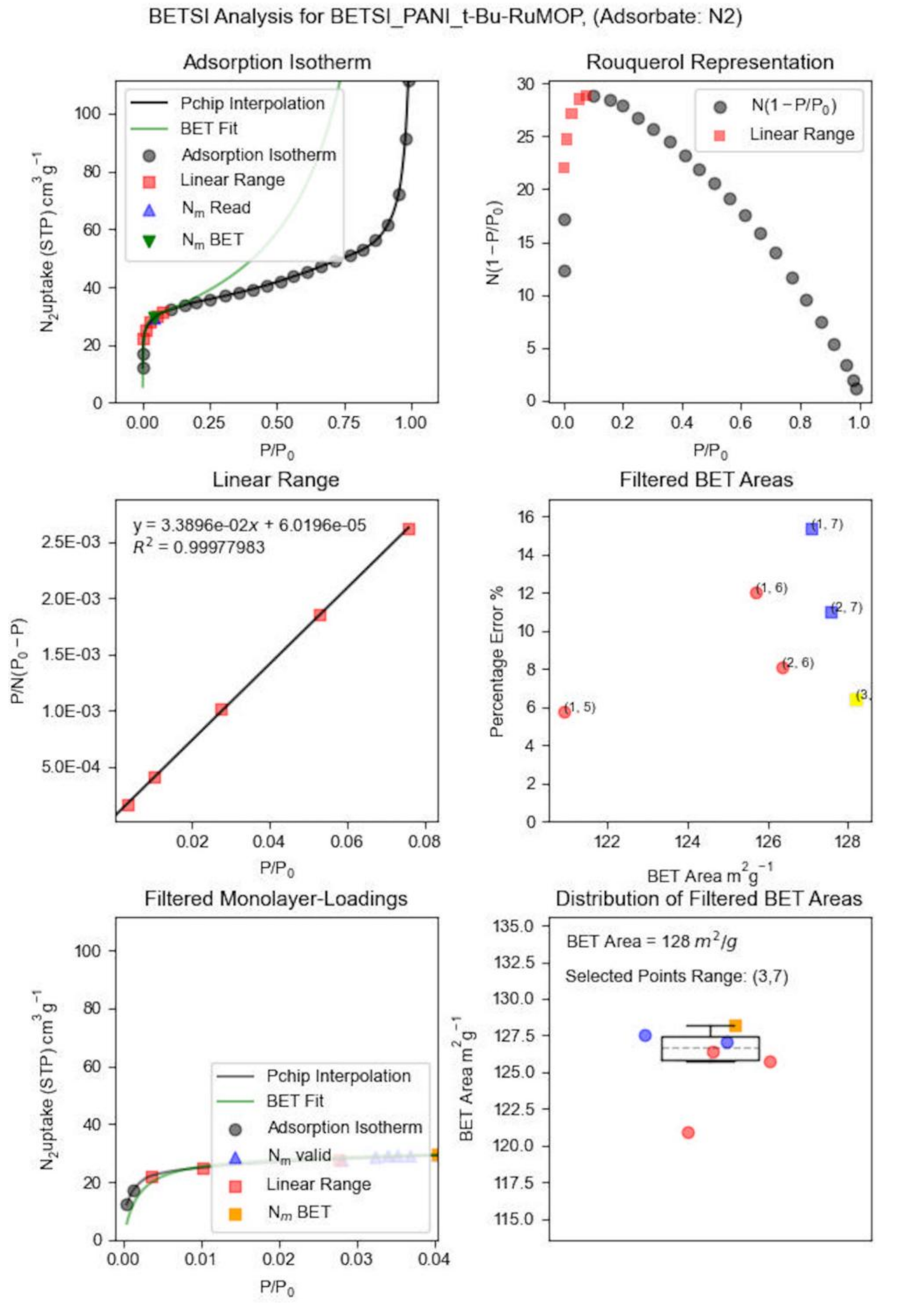

Figure S22. BETSI analysis of **PANI@*t*-Bu-RuMOP**, generated by the BETSI program.[6]

References:

1. Urbanos, F. A.; Barral, M. C.; Jiménez-Aparicio, R., Synthesis and Properties of Some Diruthenium Acetate Compounds. *Polyhedron* **1988,** *7*, 2597-2600.
2. Tayier, F.; Troyano, J.; Tokuda, S.; Wang, Z.; Haga, M.-a.; Furukawa, S., Redox-Active Ruthenium-Organic Polyhedra with Tunable Surface Functionality and Porosities. *Inorg. Chem.* **2024,** *63*, 5559-5567.
3. Chuba, T. N.; Troyano, J.; Tokuda, S.; Sánchez-González, E.; Carmona, F.; Sakaguchi, R.; Saito, K.; Kamei, K.-i.; Inose, T.; Furukawa, S., Metal-Organic Polyhedra as Intracellular Nitric Oxide Delivery Systems Towards Biomedical Applications. *ChemRxiv* **2026,** 10.26434/chemrxiv.15001526/v1.
4. Tsai, M.-D.; Chen, Y.-L.; Chang, J.-W.; Yang, S.-C.; Kung, C.-W., Sulfonate-Functionalized Two-Dimensional Metal–Organic Framework as a "Dispersant" for Polyaniline to Boost Its Electrochemical Capacitive Performance. *ACS Appl. Energy Mater.* **2023,** *6*, 11268-11277.
5. Chuang, T.-Y.; Shen, C.-H.; Tsai, H.-Y.; Wang, Y.-C.; Weng, Y.-M.; Liang, Y.-C.; Hsieh, C.-Y.; Wu, K.-C.; Chuang, C.-L.; Kung, C.-W., Mesopores in Metal–Organic Frameworks Rendering Enhanced Rate Capability of Pore-Confined Polyaniline for Supercapacitors. *ACS Appl. Mater. Interfaces* **2025,** *17*, 58314-58324.
6. Osterrieth, J. W. M.; Rampersad, J.; Madden, D.; Rampal, N.; Skoric, L.; Connolly, B.; Allendorf, M. D.; Stavila, V.; Snider, J. L.; Ameloot, R.; Marreiros, J.; Ania, C.; Azevedo, D.; Vilarrasa-Garcia, E.; Santos, B. F.; Bu, X.-H.; Chang, Z.; Bunzen, H.; Champness, N. R.; Griffin, S. L.; Chen, B.; Lin, R.-B.; Coasne, B.; Cohen, S.; Moreton, J. C.; Colón, Y. J.; Chen, L.; Clowes, R.; Coudert, F.-X.; Cui, Y.; Hou, B.; D'Alessandro, D. M.; Doheny, P. W.; Dincă, M.; Sun, C.; Doonan, C.; Huxley, M. T.; Evans, J. D.; Falcaro, P.; Ricco, R.; Farha, O.; Idrees, K. B.; Islamoglu, T.; Feng, P.; Yang, H.; Forgan, R. S.; Bara, D.; Furukawa, S.; Sanchez, E.; Gascon, J.; Telalović, S.; Ghosh, S. K.; Mukherjee, S.; Hill, M. R.; Sadiq, M. M.; Horcajada, P.; Salcedo-Abraira, P.; Kaneko, K.; Kukobat, R.; Kenvin, J.; Keskin, S.; Kitagawa, S.; Otake,

K.-i.; Lively, R. P.; DeWitt, S. J. A.; Llewellyn, P.; Lotsch, B. V.; Emmerling, S. T.; Pütz, A. M.; Martí-Gastaldo, C.; Padial, N. M.; García-Martínez, J.; Linares, N.; Maspoch, D.; Suárez del Pino, J. A.; Moghadam, P.; Oktavian, R.; Morris, R. E.; Wheatley, P. S.; Navarro, J.; Petit, C.; Danaci, D.; Rosseinsky, M. J.; Katsoulidis, A. P.; Schröder, M.; Han, X.; Yang, S.; Serre, C.; Mouchaham, G.; Sholl, D. S.; Thyagarajan, R.; Siderius, D.; Snurr, R. Q.; Goncalves, R. B.; Telfer, S.; Lee, S. J.; Ting, V. P.; Rowlandson, J. L.; Uemura, T.; Iiyuka, T.; van der Veen, M. A.; Rega, D.; Van Speybroeck, V.; Rogge, S. M. J.; Lamaire, A.; Walton, K. S.; Bingel, L. W.; Wuttke, S.; Andreo, J.; Yaghi, O.; Zhang, B.; Yavuz, C. T.; Nguyen, T. S.; Zamora, F.; Montoro, C.; Zhou, H.; Kirchon, A.; Fairen-Jimenez, D., How Reproducible Are Surface Areas Calculated from the BET Equation? *Adv. Mater.* **2022,** *34*, 2201502.
